# Adopting social health insurance in Nepal: A mixed study

**DOI:** 10.3389/fpubh.2022.978732

**Published:** 2022-12-15

**Authors:** Damaru Prasad Paneru, Chiranjivi Adhikari, Sujan Poudel, Lal Mani Adhikari, Deepak Neupane, Juli Bajracharya, Kalpana Jnawali, Kamal Prasad Chapain, Nabaraj Paudel, Nirdesh Baidhya, Ashok Rawal

**Affiliations:** ^1^School of Health and Allied Sciences, Pokhara University, Pokhara, Nepal; ^2^Department of Public Health, Indian Institute of Public Health Gandhinagar (IIPHG), Gandhinagar, India; ^3^Department of Public Health, Nobel College, Pokhara University, Kathmandu, Nepal; ^4^HERD International, Kathmandu, Nepal; ^5^School of Business, Pokhara University, Pokhara, Nepal; ^6^Department of Public Health, LA Grandee College, Pokhara University, Pokhara, Nepal; ^7^Faculty of Medical Sciences, University of Cyberjaya, Cyberjaya, Malaysia; ^8^Province Health Logistics Management Center, Gandaki Province, Pokhara, Nepal; ^9^Department of Public Health, Shaheed Krishna Sen Ichhuk Bahuprabidhik Institute, Dang, Nepal; ^10^CARE Nepal, Lalitpur, Nepal

**Keywords:** health insurance, adherence, premium, enabler, barrier, package, prepayment, Nepal

## Abstract

**Objective:**

The Social Health Insurance Program (SHIP) shares a major portion of social security, and is also key to Universal Health Coverage (UHC) and health equity. The Government of Nepal launched SHIP in the Fiscal Year 2015/16 for the first phase in three districts, on the principle of financial risk protection through prepayment and risk pooling in health care. Furthermore, the adoption of the program depends on the stakeholders' behaviors, mainly, the beneficiaries and the providers. Therefore, we aimed to explore and assess their perception and experiences regarding various factors acting on SHIP enrollment and adherence.

**Methods:**

A cross-sectional, facility-based, concurrent mixed-methods study was carried out in seven health facilities in the Kailali, Baglung, and Ilam districts of Nepal. A total of 822 beneficiaries, sampled using probability proportional to size (PPS), attending health care institutions, were interviewed using a structured questionnaire for quantitative data. A total of seven focus group discussions (FGDs) and 12 in-depth interviews (IDIs), taken purposefully, were conducted with beneficiaries and service providers, using guidelines, respectively. Quantitative data were entered into Epi-data and analyzed with SPSS, MS-Excel, and Epitools, an online statistical calculator. Manual thematic analysis with predefined themes was carried out for qualitative data. Percentage, frequency, mean, and median were used to describe the variables, and the Chi-square test and binary logistic regression were used to infer the findings. We then combined the qualitative data from beneficiaries' and providers' perceptions, and experiences to explore different aspects of health insurance programs as well as to justify the quantitative findings.

**Results and prospects:**

Of a total of 822 respondents (insured-404, uninsured-418), 370 (45%) were men. Families' median income was USD $65.96 (8.30–290.43). The perception of insurance premiums did not differ between the insured and uninsured groups (*p* = 0.53). Similarly, service utilization (OR = 220.4; 95% CI, 123.3–393.9) and accessibility (OR = 74.4; 95% CI, 42.5–130.6) were found to have high odds among the insured as compared to the uninsured respondents. Qualitative findings showed that the coverage and service quality were poor. Enrollment was gaining momentum despite nearly a one-tenth (9.1%) dropout rate. Moreover, different aspects, including provider-beneficiary communication, benefit packages, barriers, and ways to go, are discussed. Additionally, we also argue for some alternative health insurance schemes and strategies that may have possible implications in our contexts.

**Conclusion:**

Although enrollment is encouraging, adherence is weak, with a considerable dropout rate and poor renewal. Patient management strategies and insurance education are recommended urgently. Furthermore, some alternate schemes and strategies may be considered.

## Introduction

In the context of existing inequalities in health in Nepal, achieving universal access to healthcare necessitates a new form of financial hardship protection, reduction of out-of-pocket costs through subsidy or copayment, or coverage of healthcare charges. Despite the government's desperate efforts, over a quarter of the population (23%) and more than two-fifths (42%) of the population are, respectively, outside of overall preventive and treatment coverage under basic care (1, 2). The government's expenditure on health as the share of current health expenditure was below one-fourth (24.8%) and there was a high out-of-pocket expenditure for health care (58%) (3). Meanwhile, 17.4% of the population was multidimensionally poor in 2019/20 were multi-dimensionally poor (4). Furthermore, studies conducted in Kathmandu Valley in 2012 (5) and Kailali in 2019 (6) reported that 13.8 and 17.8 % of households, respectively, had experienced catastrophic health-related spending overall, whereas it was nearly 5 (4.7)% in Kaski district when age-specific (only neonatal) health problems were taken into account (7). Moreover, it was found to be 10.3% monthly at a national level, as calculated from nationally representative data from the National Living Standard Survey-2010/2011 (8). Interestingly, the study conducted in Kailali revealed the protective effect of insurance on catastrophic spending (6). The majority of people's financial incapacity is one of the main barriers to receiving healthcare in this situation. Low-income individuals and rural households could only struggle to pay for healthcare services, which would worsen their health (2) and slip into poverty and debt traps (9). On the contrary, insurance protects them from getting poorer, and thus preserves their health (6).

Various initiatives, such as the safe motherhood program in 2005 and the free health care program in 2007, have striven in the past to provide enhanced coverage of health care services in several key areas. However, those initiatives were lacking the principle of risk pooling. As a result, everyone with a stake in Nepal was concerned about safeguarding the public from catastrophic medical expenses. To reduce the financial risk associated with health care through prepayment and risk-sharing, the GoN initiated the Social Health Security Program (SHSP) in 2015, of which SHIP was a major component (10).

A seminal contribution by Arrow regarding two kinds of risks in medical care: the risk of becoming ill and its outcomes (risk of total or incomplete or delayed recovery), if not addressed with suitable insurance policies, implies a loss of welfare (11), which is very much pertinent in Nepal's context. Furthermore, it is even imperative to increase health insurance coverage as it potentially contributes to the sustainable economic growth of the nation (12). A Social Health Insurance Scheme is a mechanism that helps to mobilize resources, pool risk, and provide more access to health care services for all, particularly for the poor. This eventually helps in accessing universal health coverage (13). It is a comprehensive social contributory scheme with a subsidy to the poor and universal health coverage (UHC). It was started to ensure access to quality health services (equity and equality) and protect them from financial hardship and reduce out-of-pocket payments. GoN rolled out the first round of the SHIP in the fiscal year 2015/2016 and then registration started on 7 April 2016, in the Kailali district, followed by Baglung and Ilam on 28 June 2016. Services provisions were started in the second week of July 2015 (14) and covered all 77 districts in June 2021.

The health insurance program is a voluntary program based on family contributions. Families of up to five members have to contribute a prepayment of NPR 3,500 per year and NPR 700 per additional member. It is a cashless system for members seeking health services, information technology-based; with enrolment assistants using smartphones (15). The insureds have to choose their first service point and, can access specialized and emergency services from listed health institutions across the country on production of a referral slip from their first contact point. In 2017, only 12% of the population was covered under financial risk protection (1), and the trend was found to be increasing, i.e., 17.63% in 2022. Currently, 736 local levels are in operation, covering 18.87%% of the total population (16). According to National Health Policy 2019, diversification of equitable health insurance is mentioned in the guiding principle, and specialized services shall be made easily accessible through health insurance (15).

The adoption and future success of insurance programs are dependent on clients' perceptions and experiences regarding various attributes and their levels toward the program, such as a premium level, unit of enrollment, service management, health service benefits package, transportation coverage, and copayment levels (17). The sustainability of the program is also significantly influenced by moral hazard and other relevant elements. The SHIP has a tri-polar connection between the board, clients, members, and healthcare providers. Program adherence and continuation are affected by how each of the three components perceives the other and the program, particularly how beneficiaries and providers do. In the interim, no new studies of the SHIP had been found carried out to look at public perception, experiences, and provider views to better understand the program (18). Therefore, we aimed to assess and explore the perception, experiences, and adherence of insured and uninsured beneficiaries, and the service providers of the SHIP in early implemented districts. From this, we are able to identify the key bottlenecks and possible ways forward to adopt and sustain the program, thus starting with the service beneficiary-provider dyad.

## Materials and methods

We conceptualized the demand side perceptions from the Health Belief Model (HBM) (19, 20), whereas, for the supply side, the perceptions illustrated by Wagner (21) were considered for guidance (Supplementary Table 1). From this, we operationalized health insurance perception as perceived belief, attitude, intention, or action regarding susceptibility, vulnerability, enablers, barriers, and motivations to or against a disease or health problem or risk of acquiring them, health service or its utilization, health care provider, or insurance scheme, its premium, and benefits package (19–22).

### Study design and sites

**Figure 1:**
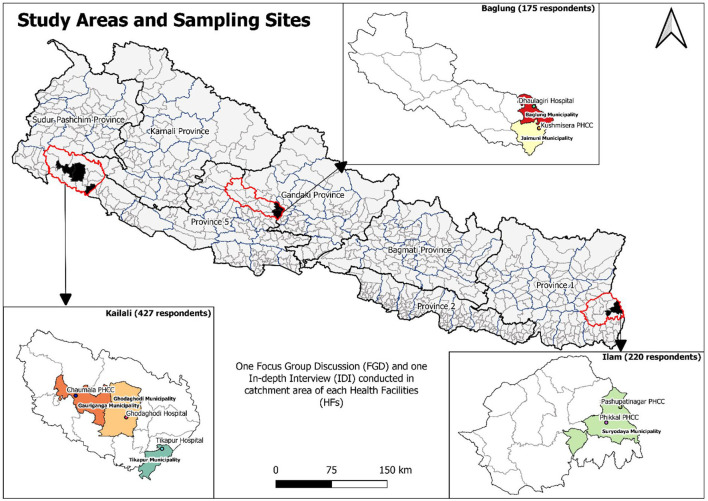
Map of the study clusters with number of respondents.

We carried out a cross-sectional, facility-based, concurrent mix methods study. Qualitative findings were explored to triangulate the quantitative results and to explore beneficiaries' and providers' different perspectives toward the Social Health Security Program (SHIP). We selected three districts—Kailali, Baglung, and Ilam—where SHIP was piloted (Figure 1).

### Sample size calculation and sampling technique

The participants were health service beneficiaries (both insured and uninsured) of the three districts and the health care providers and managers of the respective health institutions were the study participants. We selected all the insureds and the uninsured visiting the selected health facilities of three districts during the data collection periods.

The sample size for the quantitative data was determined using the formula, *n* = f(α,β) (p_1_q_1_+p_2_q_2_)/(p_1_-p_2_)^2^; after estimating service utilization among the insureds (p_1_) and the uninsureds (p_2_), proportions from (Burtibang Primary Health Care Center (PHCC) of Baglung district), (one district of study setting with a lower utilization rate) taken from PHCC records up to 12 February 2017 (p_1_ = 0.029, and p_2_ = 0.004), with 0.05 and 0.8 for alpha and beta, respectively, we obtained 407 for each group of insureds and uninsureds (total of 814), and enrolled 825 (insured-407, uninsured-418) to be safe. Three insured participants' interviews were incomplete, thus, they were excluded. We finally analyzed a sample of 822 (insured-404, uninsured-418) participants (Figure 1).

For the quantitative study, we included a proportionate sample from three districts and below-level healthcare facilities (Supplementary Table 2) to randomly include at least one hospital and one PHCC from each district. For individuals, we used the records from the Department of Health Services (DoHS) and the respective districts. Participants were selected among the insured and the uninsured in a 1:1 ratio of those visiting the health care institutions. A total of 7 FGDs (Ilam-2, Baglung-2, Kailali-3) with mixed groups of insured and uninsured people and 12 IDIs (Ilam-2, Baglung-4, Kailali-6) with SHIP focal person/manager were conducted for the qualitative study (Figure 1).

### Tools and techniques of data collection

Three pre-tested proformas, i.e., client-exit interview guideline, FGD guideline, and IDI guideline, were used to gather data from the participants from March 13 to October 21, 2020 (Figure 1; Supplementary Table 2). We tested the proformas among the patients visiting, and the health care providers of, Lekhnath community hospital in the Kaski district. We provided a two-day orientation to three undergraduate public health final year students as the enumerators. Two authors trained them with simulation exercises of interviews and discussions and also supervised them during data collection.

### Data analysis and management

Quantitative data were entered in EpiData (V 3.5.1), checked for missing values, and then imported into SPSS (V20.0), MS-Excel, and Epitools, a web-based calculator (23) for further analysis. Data was described in frequency, percentage, mean, median, interquartile range, and standard deviations. Statistics such as odds ratios and their 95 % CIs, Pearson Chi-squared test, Chi-squared test for trend analysis, and their *p*-values were used to infer the results.

Qualitative data was taken with note-keeping and checked the same day to sort out any missing. In addition, we also recorded the interviews and discussions. The qualitative data were manually analyzed with thematic analysis, progressing with codes, patterns, sub-themes, and themes. The information generated from the focus group discussion and in-depth interviews was recorded in a notebook as well as on a memory card. Recorded information was transcribed verbatim and then organized under predefined themes, for beneficiaries and service providers. We clustered the providers' codes under themes: coverage, premium, beneficiaries' behaviors, problems, occupational risks, barriers and facilitative factors, and improving strategies, whereas, for beneficiaries' codes, we deduced under knowledge, utilization, and attitude/perception toward SHIP, private sector involvement, promoting factors, improving measures, and providers' behaviors. We presented a quantitative depiction of insured and uninsured Social Health Security Program (SHIP) beneficiaries in tables and figures, as well as qualitative findings in direct verbatims and intellectual translations, and then in tables. Then we triangulated both, especially the qualitative findings for reasoning the quantitative results and to explore the perceptions and experiences.

### Ethical considerations

We obtained ethical approval from the Nepal Health Research Council, Ethical Review Board (ERB Protocol no. 835/2019 P; Ref no. 1691 dated 24 January 2020), and respective health institutions and municipality offices of the selected districts. All participants were informed about the study objectives and written informed consent from literate people and verbal from illiterate people were taken before the interview.

## Results

The quantitative, descriptive, and inferential results and qualitative findings obtained from themes and sub-themes are presented under different sub-headings.

### Socio-demographic and economic characteristics

More than half of the participants were from the Kailali district, followed by 26.8% from Ilam and 21.3% from Baglung. The majority of them (55.0%) were women, and men respondents outnumbered women in Kailali. The highest proportion of the participants was aged 20–29 years (30.0%), and the mean age was 37.4 (±14.1) years. Almost 4% were adolescents and 8% were elderly participants. The median monthly household income was NPR 20,000 (10,000–35,000) (US $ 1 = NPR 120.51) (24) with half of the participants having a monthly income below NPR 20,000(0–350,000). A majority (53.3%) of the participants were Brahmin followed by Janajati (22.1%) and others, as depicted in Table 1.

**Table 1:** Socio-economic characteristics of participants (*n* = 822).

**Variables**		**Districts**			**Total**
**Name categories**		**Ilam**	**Baglung**	**Kailali**	
		220 (26.8)	175 (21.3)	427 (51.9)	822 (100.0)
Gender	Male	91 (11.1)	64 (7.8)	215 (26.2)	370 (45.0)
	Female	129 (15.7)	111 (13.5)	212 (25.8)	452 (55.0)
Age (yrs)	< 20	6 (0.7)	8 (1.0)	18 (2.2)	32 (3.9)
	20–29	59 (7.2)	39 (4.7)	149 (18.1)	247 (30.0)
	30–39	73 (8.9)	36 (4.4)	131 (15.9)	240 (29.2)
	40–49	39 (4.7)	30 (3.6)	53 (6.4)	122 (14.8)
	50–59	27 (3.3)	35 (4.3)	53 (6.4)	115 (14.0)
	≥60	16 (1.3)	27 (3.3)	23 (2.8)	66 (8.0)
	Mean (SD)	37.3 (12.4)	42.1 (15.9)	35.4 (13.6)	37.4 (14.1)
	Median (Min-Max)	35 (16–77)	40 (17–87)	33 (16–86)	35 (16–87)
Monthly income*	Median (Q_1_–Q_3_)	20,000 (10,000–30,000)	20,000 (12,000–40,000)	20,000 (10,000–30,000)	20,000 (10,000–35,000)
Ethnicity	Brahmin/ Chhetri	79 (9.6)	142 (17.3)	217 (26.4)	438 (53.3)
	Janajati	128 (15.6)	25 (3.0)	29 (3.5)	182 (22.1)
	Dalit	11 (1.3)	5 (0.6)	45 (5.5)	61 (7.4)
	Madhesi/ Tharu	0 (0.0)	0 (0.0)	100 (12.2)	100 (12.2)
	Others	2 (0.2)	3 (0.4)	36 (4.4)	41 (5.0)

*In NPR (USD 1 = NPR 120.51 as per NRB, July 1, 2020).

### Awareness, enrollment, and adherence to a health insurance program

Positive perception towards SHIP is in progressive way, and the enrollment rate was also found to be in an increasing trend except in 2019. Meanwhile, the dropout rate is also increasing, nearly at the rate of one in every ten (162/342, 47.4% for 2 times; 131/342, 38.3% for ≥3 times; difference, −9.1%) as calculated from the total registered and insureds of 342 in three districts in 6 years (2014–2019) (**Figure 3**).

**Figure 2:**
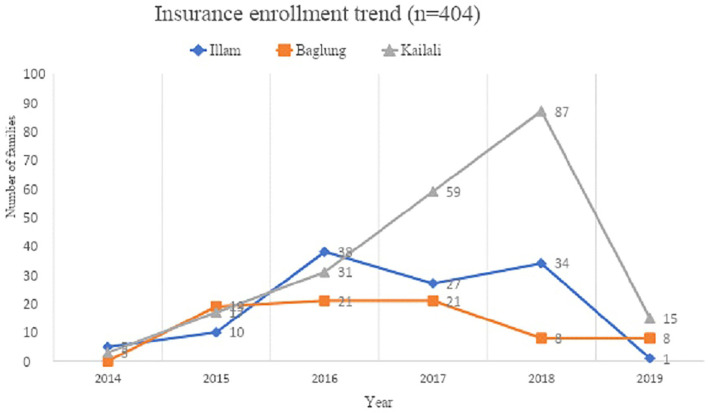
Trend showing year-wise enrollment of insureds in three districts (Pearson Chi-squared (df), 34.29 (4); *p* < 0.001; Chi-squared for slope (df), 8.69 (1); *p*, 0.003; slope, −0.007; Chi-squared (df) for non-linearity; 25.59 (3), *p* < 0.001; After removing 2019 data, Chi-squared (df) for non-linearity; 23.66 (2), *p* < 0.001).

**Figure 3:**
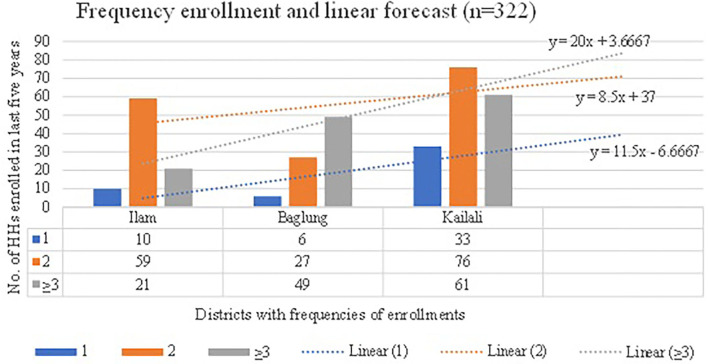
Frequencies of HHs registered in insurance program and forecasting with equations (Pearson Chi-squared (df), 7.01(1); *p*, 0.008; Chi-squared for slope (df), 7.79 (1); *p*, 0.005; slope, 0.007; Chi-squared (df) for non-linearity, −0.778 (0); p,1).

The perception of the need/importance of SHIP differed significantly between insured and uninsured beneficiaries (*p* < 0.001) (Table 2). In line with this, the high level of awareness of SHIP is reflected in the trend analysis, where year-wise enrollment trends of the family registered in health insurance scheme in three districts during 2014–19 showed differences although the trend, is increasing, in all districts, except for 2019 (Figure 2). Chi-square for linear trend analysis showed that year-wise enrollment in three districts was non-linear [X^2^ (df), 25.6(3); *p* < 0.001] and remained unchanged even after excluding 2019 data [X^2^(df), 23.66(2); *p* < 0.001] (Figure 2). However, the frequencies of registered HHs showed a linear trend [X^2^(df), −0.778 (0), *p* = 1; slope = 0.007, *p* = 0.005] (Figure 3). The forecast model and equations show that a range of nearly 9–20 HHs, from 2 to 3 or more times registered respectively, will be increased when adding a next district (Figure 3). However, it may differ according to the district population and other socio-demographic and implementation variables. Respondents claimed that the enrollment in SHIP was encouraging and might have reached more than half of the population. These claimants are consistent with qualitative findings from the service providers. The enrollment trend (Figure 3) forecasted from our data was obvious as it was further explained during the interviews. However, a few participants also reflected a negative perception of SHIP.

*I got enrolled in the SHIP since its inception (2015/16 AD)*.

*-A beneficiary of Phikkal PHCC, Ilam*

*If we have an insurance card with us, we can get services from every government health institution up to 50,000*.

*-A beneficiary of Chaumala PHC, Kailali*

*SHIP saves health care costs and it is extremely useful when someone has financial hardships. If health insurance is done, treatment can be done at a minimal cost*.

*-A beneficiary of Tikapur PHC, Kailali*

*We did not enroll in SHIP due to a lack of money. We are not sick, so why do we need SHIP*.

*-An uninsured beneficiary of Kushmishera PHCC, Baglung*

*Stakeholders must encourage the uninsured to get enrolled in SHIP*.

*-A beneficiary from Tikapur hospital, Kailali*

*People are mostly unaware of the insurance and those who are insured, have no proper idea in the process of getting enrollments and service use under SHIP*.

*-A beneficiary from Tikapur hospital, Kailali*

**Table 2:** Associative demand-side perceived factors among the beneficiaries.

**Variable description**		**Insurance status**		**Total**	***p*-value**	**UOR (95 % CI)**
		**Insured**	**Uninsured**			
Perception of premium	Expensive	125 (30.9)	121 (28.9)	246 (29.9)	0.53	-
	Not Expensive	279 (69.1)	297 (71.1)	576 (70.1)		
Perceived differences in service availability	Yes	301 (74.5)	206 (49.2)	507 (61.6)	< 0.001**	3 (2.23–4.03)
	No	103 (25.5)	212 (50.8)	315 (38.4)		Ref
Perceived importance of SHIP	Important	365 (91.0)	259 (64.1)	624 (75.9)	< 0.001**	5.74 (3.91–8.44)
	Not Important	39 (9.0)	159 (35.9)	198 (24.1)		Ref
Perception of the family as a unit	Important	362 (89.6)	272 (65.0)	634 (77.1)	< 0.001**	4.62 (3.17–6.74)
	Not Important	42 (10.4)	146 (35.0)	188 (22.9)		Ref
Perception of SHIP for underprivileged	Important	385 (95.2)	320 (76.5)	705 (85.7)	< 0.001**	6.20 (3.71–10.36)
	Not Important	19 (4.8)	98 (23.5)	117 (14.3)		Ref
Perception of annual renewal rule/system	Important	278 (68.9)	223 (53.4)	501 (60.9)	< 0.001**	1.92 (1.45–2.56)
	Not Important	126 (31.1)	195 (46.6)	321 (39.1)		Ref
Perception of the benefits package	Effective	197 (48.7)	109 (26.0)	306 (37.2)	< 0.001**	2.69 (2.01–3.61)
	Not Effective	207 (51.3)	309 (74.0)	516 (62.8)		Ref
Perception of referral system	Effective	150 (37.1)	96 (22.9)	246 (29.9)	< 0.001**	1.98 (1.46–2.68)
	Not Effective	254 (62.9)	322 (77.1)	576 (70.1)		Ref

*Statistically significant at p < 0.05; **Statistically significant at p < 0.001.

The reason for increased enrollment is further supported by the fact that the service providers also mentioned that it may have started as a result of more surgery facilities and a better referral system.

*Emergency and referral services are provided from this hospital, and mostly insured patients attend PHCC and are referred to a higher center. Enrolled people have received services provided under SHIP. In the meantime, more than 60% of the insured population were not satisfied with service management under SHIP*.

*-A hospital SHIP focal person, Baglung*

*Some people received services for the first time, and thereafter they seldom received services due to the perceived poor quality of service and its management*.

*-A beneficiary from Dhaulagiri hospital, Baglung*

*If there is timely reimbursement to the service-providing institutions, then the satisfaction level among beneficiaries will be high, and as a result, coverage will also increase*.

*-A service provider, Ghodaghodi hospital, Kailali*

Furthermore, some quantitative findings of the supply-side revealed that the insureds received twice as many timely follow-up services (OR = 2.3; 95% CI, 1.2–3.0) as their counterparts (*p* < 0.001). This might have prevented a further decrease in the dropout rate.

### Service quality and utilization

Poor service utilization was associated with physical distances of more than 30 min that it took to reach the health facility. In addition, the poor quality of the services was also a co-factor.

A significant difference was observed in the time taken (with a cut-off of 30 min) to reach HFs, between the insureds and the uninsureds (*p* = 0.023, Supplementary Table 5), specifically, that of Kailali district (*p* = 0.013, Supplementary Table 5). Moreover, accessibility (OR = 74.4; 95% CI, 42.5–130.6) and service utilization (OR = 220.4; 95% CI, 123.3–393.9) both were very strongly associated with the insured beneficiaries (**Table 4**), however, this may need further cautious interpretation. The availability of emergency services and SHIP services managed in HFs (all *p's* > 0.05, Table 3) did not differ between the two beneficiary groups. Similarly, the distance between the health facility (HF) and home (5 km or less) (*p* > 0.05, Table 3) and time taken (with a cut-off of 30 min) to reach HFs, in case of Ilam and Baglung districts, did not differ between the two types of beneficiaries (*p* > 0.05, Supplementary Table 5). The quality factors were explored during the interviews with beneficiaries and the service providers.

*Some people received service for the first time, and thereafter they seldom received services due to the poor quality of service and its management*.

*-A beneficiary, Kailali*

*I visited a health institution three times for the treatment of a single disease but didn't receive any treatment. At different times, I faced a shortage of medicine, equipment, or health personnel*.

*-An insured beneficiary, Ilam*

*Initially, it was very good but, in the middle, there was a scarcity of medicines, which resulted in a decrease in the number of insured people. However, the coverage is increasing now*.

*-Service provider, Tikapur hospital, Kailali*

**Table 3:** Associative supply-side quality and availability factors of insurance providing HFs.

**Variable description**		**Insurance status**		**Total**	***p*-value**	**UOR (95 % CI)**
		**Insured**	**Uninsured**			
Distance between health institution and home	>5 Km	316 (78.2)	339 (81.1)	655 (79.7)	0.30	-
	≤ 5 Km	88 (21.8)	79 (18.9)	167 (20.3)		
Follow-up services/visits	Yes	253 (62.6)	190 (45.5)	443 (53.9)	< 0.001**	2.01 (1.52–2.65)
	No	151 (37.4)	228 (54.5)	379 (46.1)		
Timely follow-up service received	Yes	246 (60.9)	170 (40.6)	416 (50.6)	< 0.001**	2.27 (1.17–3.0)
	No	158 (39.1)	248 (59.4)	406 (50.4)		
Availability of emergency services	Yes	342 (84.6)	347 (83.0)	689 (83.9)	0.52	-
	No	62 (15.4)	71 (17.0)	133 (16.1)		
Proper service management	Yes	262 (64.9)	286 (68.4)	548 (66.7)	0.27	-
	No	142 (35.1)	132 (31.6)	274 (33.3)		

*Statistically significant at p < 0.05; ** Statistically significant at p < 0.001; ^#^Fisher Exact Test.

### Service coverage, accessibility, and availability

Since perception toward the SHIP was found to be positive, which might have been associated with the availability and as a result, increased coverage. Consideration of an alternate benefits package that may include the medicines for major Noncommunicable Diseases (NCDs) along with kidney-related problems and related essential medicines was emphasized by the beneficiaries.

From the demand side, quantitative findings indicated that service accessibility (OR = 74.4; 95% CI, 42.5–130.6) and service utilization (OR = 220.4; 95% CI, 123.3–394.0) were highly associated among the insured beneficiaries as compared to their counterparts. Among the types of services, general health checkups (OR = 109.8; 95% CI, 64.0–188.3), emergency services (OR = 80.8; 95% CI, 19.8–329.6), and referral services (OR = 81.4; 95% CI, 11.2–589.7) were also strongly associated with insured beneficiaries (Table 4). These strong associations are further verified by the perceptions of beneficiaries toward SHIP (Table 2). Availability of services was differently perceived three times (OR = 3; 95% CI, 2.23–4.03) and benefits package was perceived more than two times (OR = 2.69; 95% CI, 2.01–3.61) as “effective” among the insured beneficiaries compared to their counterparts.

*I had undergone surgery for gallstones and was satisfied with the services and the referral system*.

*-An insured beneficiary, Pashupatinagar PHCC, Ilam*

Although the demand-side factors favored the insureds, the uninsured and some insured claimed that the coverage of the SHIP is low and services are not satisfactory.

*My mother is suffering from a kidney-related problem, but the treatment is not available through SHIP*.

*- A beneficiary, Tikapur PHCC, Kailali*

*We can't get any of the services that we are seeking. Very few services are included in SHIP*.

*-A beneficiary, Pashupatinagar PHCC, Ilam*

*All medicines are not available; all diseases are not treated; and there is no coverage for expensive medicines*.

*-A beneficiary, Kushmisera PHCC, Baglung*

*The services should not be limited to minor diseases only. They should be more focused on treating major NCDs, too*.

*-A beneficiary from Phikkal PHCC, Ilam*

**Table 4:** Associated demand-side factors of insurance providing health facilities.

**Variable description**		**Insurance status**		**Total**	***p*-value**	**UOR (95 % CI)**
		**Insured**	**Uninsured**			
Accessibility	Yes	389 (96.2)	108 (25.8)	497 (60.5)	< 0.001**	74.4 (42.5–130.6)
	No	15 (3.8)	310 (74.2)	325 (39.5)		Ref
Services utilization	Yes	367 (90.8)	18 (4.3)	385 (46.8)	< 0.001**	220.42 (123.32–393.99)
	No	37 (9.2)	400 (95.7)	437 (53.2)		Ref
**Types of services received**
General health checkup	Yes	336 (83.1)	18 (4.3)	354 (43.0)	< 0.001**	109.82 (64.02–188.31)
	No	68 (16.9)	400 (95.7)	468 (57.0)		Ref
Emergency service	Yes	113 (35.7)	2 (0.5)	115 (13.9)	< 0.001**^#^	80.76 (19.79–329.55)
	No	291 (64.2)	416 (99.5)	707 (86.1)		Ref
Referral services	Yes	66 (16.4)	1 (0.3)	67 (8.2)	< 0.001**^#^	81.42 (11.24–589.74)
	No	338 (83.6)	417 (99.7)	755 (91.8)		Ref

*Statistically significant at *p* < 0.05; **Statistically significant at *p* < 0.001; ^#^Fisher Exact Test.

### Beneficiary-provider communication and behaviors

A non-significant difference in experiencing the unfriendly behavior of health workers was observed between two beneficiaries (*p* = 0.626) (Table 5). However, during the interviews, beneficiaries revealed their dissatisfaction with the healthcare provider's behaviors (Table 4).

*I am quite unsatisfied with the services, as the service provider's way of dealing with the public was not appropriate. Therefore, I discontinued the SHIP program*.

*-A discontinued beneficiary from Pashupatinagar PHCC, Ilam*

*When we go to the hospital, they ignore the patients and say there is no medicine*.

*-A beneficiary of Dhaulagiri hospital, Baglung*

*Service providers pay less attention to the insured people than the uninsured, and services are not provided on time to the insured ones*.

*-An insured beneficiary, Ghodaghodi hospital, Kailali*

**Table 5:** Experiences of the insured and uninsured beneficiaries toward the SHIP and providers.

**Items (multiple responses)**	**Total^#^**	**Insurance status**		***p*-value**
		**Insured**	**Uninsured**	
IEC materials	229	136 (59.4)	93 (40.6)	< 0.001**
Expanding service package	130	88 (67.7)	42 (32.3)	< 0.001**
Availability of human resources	110	72 (65.5)	38 (34.5)	< 0.001**
Public awareness about SHIP	92	51 (55.4)	41 (44.6)	0.200
Services for underprivileged	89	56 (62.9)	33 (37.1)	0.005**
Availability of laboratory services	79	44 (55.7)	35 (44.3)	0.220
Free/low-cost services	72	51 (70.8)	21 (29.2)	< 0.001**
Improving service management	65	47 (72.3)	18 (27.7)	< 0.001**
Household visit by enrollment staff	59	32 (54.2)	27 (45.8)	0.417
Waiting time	179	101 (56.4)	78 (43.6)	0.027*
Service delivery	167	89 (53.3)	78 (46.7)	0.230
Availability of medicine /services	154	88 (57.1)	66 (42.9)	0.027*
Crowding	123	56 (45.5)	67 (54.5)	0.383
Providers' behaviors	121	57 (47.1)	64 (52.9)	0.626
Coverage of health problems	107	48 (44.9)	59 (55.1)	0.341
Enrollment process	106	49 (46.2)	57 (53.8)	0.519
Public trust in services	98	46 (46.9)	52 (53.1)	0.641
Available human resources	67	33 (49.3)	34 (50.7)	0.985
Financial status	49	22 (44.9)	27 (55.1)	0.539

*Statistically significant at p < 0.05; **Statistically significant at p < 0.001; ^#^Multiple responses; figures in parentheses are %ages.

It is not all the healthcare provider's behaviors, but a few having poor communication skills that might be creating problems, as, on average, no difference was experienced by the two beneficiaries. In addition, the Healthcare Provider (HCP) further clarified that the high expectations of the beneficiaries and poor patient management might be creating some problems (Table 6).

*Insured people sometimes feel irritated waiting in a queue to receive services. Both insured and uninsured patients face some problems during high patient flow*.

*-A service provider, Pashupatinagar PHCC, Ilam*

*It is only their feelings, but there are no problems they have to face. He further added, “Both the insured and uninsured have to wait for their checkup time to come in queue if the patient flow is high.”*

*-A service provider, Ghodaghodi hospital, Kailali*

*HCP provided services to infectious patients without personal protective equipment (PPE) such as gloves and masks*.

*-A service provider, Pashupatinagar PHCC, Ilam*

*Dealing with the rude behaviors of patients, when their high expectations were not met, and the existing state of service delivery creates an unusual and unpleasant environment. Many patients are unaware of the SHIP and come to the hospital expecting to receive all free services. If their services are not covered by insurance, they become annoyed and badly scold the service providers*.

*-A service provider, Tikapur hospital, Kailali*

**Table 6:** Summary findings from service providers (qualitative).

**SN**	**Theme/Pattern**	**Summary (intellectual translation)**
1	Coverage of SHIP	Service providers and managers reported that there was almost 60–80% coverage of SHIP among the population in Illam, Baglung, and Kailali. Insurance agents of the respective wards in different districts facilitated the beneficiaries for SHIP enrollment. However, their limited mobility in the community had made a steady increment in SHIP coverage. Although there was incremental acceptance of SHIP was observed in the initial days, the declining trend was observed in subsequent years due to the limited availability of medicines and services under SHIP. It was noted that almost all people with chronic diseases have made health insurance.
2	Perceived premium and benefit package	The existing premium was perceived to be appropriate and affordable. Coverage of the service has been improving; however, the availability of limited services sometimes makes questionable about the premium. Services under SHIP were perceived as cost-effective and mostly used by insured people. Insured people who had hypertension mostly utilized the services of SHIP. Community acceptance was improved with 80–90% of people being satisfied with SHIP. Nevertheless, community acceptance of SHIP in Kailali was low because of the lower level of awareness among the population. The referral center was limited to a few hospitals only. There were problems in accessing and purchasing the equipment and medicines. Patients referred to higher centers have to be in the queue for care in higher health care institutions because of this, some insured people also discontinued. The annual renewal system of SHIP was not perceived to be user-friendly due to which there was poor adherence and a high proportion of drop out.
3	Providers' perception of beneficiaries' behavior	There was an affirmative perception of the insured and uninsured people regarding SHIP. Insured people have good availability and coverage of care. Insured people perceived that the services must be made available even from private institutions. Beneficiaries become disappointed when the uninsured got limited services to those insured beneficiaries and they feel irritated waiting in a queue to receive services. In addition, sometimes it is difficult to avail of services in time, and the referral system is also felt hectic.
4	Problems faced by insured and uninsured beneficiaries availing services	Limited availability of services from SHIP-implemented health institutions, long waiting times for their checkup/health care, and perception of difficulty in dealing with a hectic referral system. In the meantime, there were no verbal complaints as well as written feedback from the public and beneficiaries in regard to the problems faced.
5	Providers' perception of underprivileged	Effective delivery of services at all times, waiving the premium and renewal amount for insurance, making the SHIP compulsory for people with low SES, and providing awareness at the household level through mass campaigns or frequent visits made by the insurance agents may be useful to make insurance coverage of the underprivileged population.
6	Problems faced by service providers	Limited human resources, service packages, and financial resources regarding patients' expectations, nagging from the public, delayed reimbursement of expenditures made, and dealing with the rude behaviors of some people at the time-of-service delivery were the major problems encountered by service managers and providers in the study districts.
7	Occupational risk experienced by HCPs	The unavailability of the PPEs was experienced as an occupational risk among the SHIP providers and they also added that some patients' behaviors put them at occupational risk.
8	Facilitative factors of the SHIP program	Regular monitoring of the program, regular supply of services and medicines, good inventory management, monthly progress reporting, review meetings, and distribution of responsibilities among the staff facilitated the management of SHIP.
9	Ways to reduce the barriers (by HCPs)	Timely reimbursement of expenditures made for health care, promoting service provider-community bonding, use of technology and Apps, and mobilizing the trained human resources are useful to strengthen SHIP.
10	Measures to improve coverage, accessibility, and implementations modalities of SHIP	Mobilization of insurance agents, publicizing the program highlights among beneficiaries through different individual, group, and mass methods, expansion of services to both the public and private institutions, and development the effective referral mechanisms gradually improve the coverage of services and effective implementations of SHIP.

In addition, some service providers are also lacking the necessary personal protective equipment (PPEs).

### Premium, risk pooling, renewal, and benefits package

The premium for insurance was not found to be significantly different among the two groups of beneficiaries. However, it was perceived that the program is important for the underprivileged. The underprivileged, on the other hand, are less health-conscious and needed to be enrolled through alternative means even if they cannot afford the premium.

Even though perception regarding the SHIP premium did not differ significantly between the two beneficiary groups (*p* = 0.53), perception toward other related variables was found to be highly associated with the SHIP. When compared to their counterparts, insured beneficiaries perceive the importance of annual premium renewal as nearly two times higher (OR = 1.96; 95% CI, 1.45–2.56), the perceived benefit package as nearly three times higher (OR = 2.69; 95% CI, 2.01–3.61), and the perceived referral system as nearly two times higher (OR = 1.98; 95% CI, 1.46–2.68) (Table 3). In addition, the experience of expanding the benefits package significantly differed between the two beneficiaries (*p* < 0.001) (Table 5).

*There was once a villager whose treatment cost for a disease was about Rs 50,000(US $1* = *NPR 120.51)* (24)*, of which about Rs 40,000 of that cost was borne by the insurance. This incident increased the enrollment and motivated people toward SHIP*.

*-An insured beneficiary from Phikkal PHCC, Ilam*

Similarly, the perception of both families as a unit and underprivileged (*p's* < 0.001) (Table 2) were significantly different in the two beneficiary groups, with higher proportions among the insureds. However, mixed findings were revealed during the interviews.

*Family as a unit is very supportive in healthcare; however, it can be a problem for a joint family with more than ten members*.

*-A beneficiary of Tikapur PHC, Kailali*

*It is a total waste of money. My family doesn't get the chance to utilize that much*.

*-A beneficiary of Tikapur PHC, Kailali*

Although there was a significant difference in experiences of free or low cost SHIP between the two groups (*p* < 0.001) (Table 5), some families only revealed their willingness to pay a threshold of 1000 NPR during the interviews (Table 7).

*The premium is a little expensive and it would be more appropriate if the amount was around Rs 1,000 (US $8.30) instead of paying Rs 3500 ($34.14) annually* (USD 1 = NPR 120.51) (24).

*-A beneficiary from Pashupatinagar PHCC, Ilam*

**Table 7:** Summary findings of the beneficiaries (insured and uninsured) (qualitative).

**SN**	**Theme/Pattern**	**Summary (intellectual translation)**
1	Knowledge about the SHIP	The proportion of the population knowing SHIP ranged from a few to a 100%. A Participant from Baglung reported that all of them had the knowledge and knew few too many participants from Kailai and Ilam had heard about SHIP. The majority of the insured participants were more ere knowledgeable than the uninsured while some of the insured participants did not have comprehensive knowledge about SHIP.
2	Utilization of SHIP	Enrollment in SHIP was encouraging in all the study districts as almost 50–90% population were enrolled in the program. The new enrollments each year from the inception of the program have been increasing; however, the dropout among the insured population was also continued. Among the uninsured population, they remain uninsured because of a lack of awareness, and financial limitations and some felt that less importance of the program. Both the insured and uninsured people had received the services from SHIP-implemented health care institutions. For those who discontinued the SHIP, the major reasons for the discontinuation were the poor perceived quality of care, limited availability of care, and health workers' unfriendly behaviors.
3	Perception of SHIP	There was a mixed perception regarding the premium amount charged for SHIP enrollments. The majority of the beneficiaries opined that it is appropriate and affordable while some others stated that it is expensive. It is better if the premium cost is borne by the government in the case of marginalized people or those who face financial hardship. Participants from Baglung stated, “If we fall sick, then there is an increase in expenditure for treatment, so SHIP is good while in case of no sickness, it is a waste of money.” Household/family size as the unit beneficiary for SHIP was perceived to be a good idea. Nonetheless, it's a problem for a family with members of more than 10. The inclusion of members up to 6 with a minimum premium could cover the grand parent's insurance which may become a useful model in Nepal. Coverage of care under SHIP has been increasing since its inception with 70-80 % population in SHIP-implemented areas being covered for this scheme while dropout/discontinuity was also reported among large populations. Door-to-door visits made by insurance agents made it possible to improve enrollments in SHIP. Since the nature and the type of services covered under the SHIP do not meet the needs of health care, patients have to seek care from other health care institutions which limit the coverage. In the meantime, the uninsured claimed that the coverage of the SHIP is low and services are not satisfactory. Participants from Ilam opined that the management of services has been improving gradually whereas it was reported to be poor in Baglung and Kailali. Shortage of medicines and equipment, limited availability of service items, poor smoothing in service delivery, long waiting time, non-coverage of expensive medicines, and unfriendly renewal system of SHIP have made limiting attractions toward the program. Therefore, the benefits package under the SHIP was perceived to be low and expressed the need for expansion.
4	Private sector involvement	The private sector's involvement in SHIP was negligible except in areas where the SHIP was implemented in Private hospitals.
5	Perceived promoting factors	Community and group-based awareness programs, spreading the information to the peripheral level, the addition of service packages, placement of citizen charter, orienting the benefits of service packages under SHIP, mobilizing FCHVs to inform people, engaging local authorities, timely follow up for renewal, timely delivery of services and mobilizing insured beneficiaries to motivate the public might have promoted the acceptances of SHIP.
6	Suggestions to improve SHIP	Effective mobilization of insurance agents, an extension of services to private institutions, periodic monitoring continuously, tracking of the service delivery mechanism, further addition of services into existing benefit packages, extending services for NCDs, availing services from all service points irrespective of the first contact point and developing the user-friendly referral mechanisms are useful in strengthening SHIP. Similarly, advertising of SHIP program in wider dimensions, paying equal attention to both the insured and uninsured people, training the health workers, and social leaders, and orienting the local people could promote the SHIP. Furthermore, the provision of insurance free of cost for an underprivileged population with increasing awareness and improving the management of health institutions for effective delivery of services are also useful strategies for the promotion of SHIP.

The coverage of one lakh by insurance was found satisfactory among the beneficiaries. However, on one hand, it seems that less risk perceiving people may repel the program, such by assuming that it would be a waste when there was nobody sick from their family, and on the other hand, those unable to pay are needed to be enrolled through other safety nets, such as by local bodies or the similar.

*It is good that NPR per family pertains to coverage of 1 lakh*.

*- A beneficiary, Dhaulagiri Hospital, Baglung*

*The package is incomplete. I once extracted a tooth, but there is no provision for replacing that tooth with a new one*.

*-A beneficiary, Tikapur hospital, Kailali*

*If we fall sick, then there is an increase in expenditure for treatment, so SHIP is good, while in the case of no sickness, it is a waste of money*.

*-A beneficiary, Kushmisera PHCC, Baglung*

*A respective municipality must enroll underprivileged groups into SHIP rather than providing them with relief funds*.

*-A service provider, Ghodaghodi hospital, Kailali*

### Barriers, facilitative factors, and ways forward

Since public trust toward the scheme is similar in both groups, the program may be scaled-up provided that people are aware of the availability and affordability of medicines, better managed with regular human resources, and service packages can be modified as per the population's health needs. Integration with other programs and further technology and user-friendly improvements are also important.

In quantitative findings (Table 5), experiences of difficulty in the enrollment process (*p* = 0.519), limited human resources (*p* = 0.985), overcrowding (*p* = 0.383), the poor financial status of relevant health facilities (*p* = 0.539), and HH visits by enrollment staff (*p* = 0.417) remained non-significant in the difference between two groups. However, people from the rural parts of the respective catchment areas are still unaware, and the public perception is not favorable (Table 7).

*It has neither covered a large population nor has the awareness regarding SHIP reached the rural parts of Nepal*.

*-A beneficiary from Pashupatinagar PHCC, Ilam*

*Change in public perception is much needed*.

*-A beneficiary, Pashupatinagar PHCC, Ilam*

*Stakeholders must encourage the uninsureds to get enrolled in SHIP*.

*-A beneficiary from Chaumala PHCC, Kailali*

Another barrier is the non-transferability of the insurance scheme. They are not getting the benefits outside their catchment areas (Table 7).

*The people who are currently living in Kathmandu but enrolled in Phikkal PHCC cannot avail of service under SHIP and they cannot come to Ilam for renewal or referral cards. Due to such conditions, the dropout rate has also increased and motivation for SHIP has decreased*.

*-An insured beneficiary from Phikkal PHCC, Ilam*

*Information should be reached to all sections of the population, including marginalized people*.

*-A beneficiary, Pashupatinagar PHCC, Ilam*

To further expand the program, integration with other programs such as the elderly allowance card, aligning with the Female Community Health Volunteer Program, and local governments are important. Beneficiaries' experiences differed in information education and communication materials significantly (*p* < 0.001) (Table 5), which shows that they can be helpful to create awareness for the program extension.

*Many people still do not have awareness about the SHIP. Community acceptance is increasing though. Some elderly people expect the SHIP services to come with an old age allowance card*.

*-A service provider, Chaumala PHCC, Kailali*

Although there was a significant difference in experience in improving service management (*p* < 0.001) and availability of human resources (*p* < 0.001) between the two groups, public trust toward the program did not differ significantly (*p* = 0.641) (Table 5).

*Regular monitoring of the program, regular supply of services and medicines, good inventory management, monthly progress reporting and review meetings, distribution of responsibilities among the staff, and contingency meetings for the immediate issues facilitated the management of SHIP. Moreover, an online management system, the use of a mobile app, improving public awareness to reduce rumors, and effective mobilization of insurance agents at the grass-root level are also important*.

*-Service providers from Kushmisera PHCC and Dhaulagiri hospital, Baglung*

*Spreading awareness through FCHVs and working in coordination with local bodies could improve the coverage, and the health institutions could discharge their responsibilities on par with their job descriptions*.

*-A service provider, Ghodaghodi hospital, Kailali*

## Discussion and prospects

We discuss the enrollment, service utilization, premium and risk pooling, behavioral aspects, barriers, and facilitative factors of SHIP. Some alternate but relevant strategies have also been argued in national and international contexts.

### Awareness, enrollment, and adherence

This theme of SHIP addresses and supports the breadth of universal health coverage (UHC). Awareness regarding the SHIP was increasing but adherence was poor, i.e., dropping down but slower than enrollment. As our forecasting equation shows, a minimum of around nine to 20 HHs may be increased by rolling up with an additional district (Figure 3). It looks obvious when observed that mandatory enrollment and providing up to 60% subsidies to all insured people took 20 years to cover 100 % of the population in Mongolia (25). It could take a long time, or it could never reach universal enrollment, as in our case, where a voluntary mechanism is in place.

Furthermore, a scaled scenario of the Pokhara metropolitan Kaski district also showed that the dropout proportion is as high as one-fifth, as revealed by Sharma et al. (26), which is nearly double our study (9.1%). This may show in high dissatisfaction among families, especially among urban dwellers. Another similar study carried out in Bardiya, Chitwan, and Gorkha showed that enrollment increased from 1% in 2016 to 11% in 2019, and dropout decreased from 67% in 2016 to 38 % in 2018. However, dropout remained a key challenge for the sustainability of the health insurance program in Nepal (27).

The HI Board mentioned some challenges like it is unable to provide free-of-cost services to poor families due to the absence of poverty cards for the poor; supply-side barriers, i.e., the availability of drugs, diagnostic services, and doctors are significant drivers of enrolment and service utilization; and still a lack of enrolling all the targeted population due to low level of awareness (28). Despite this, the survey in the same districts showed a majority of the participants (90%) had heard of the insurance scheme and believed that enrolling in it would be a proper way to minimize their financial burden (29).

The reasons for such a low enrolment rate must be sought in the limited capacity of schemes that are based on only one health facility (public schemes) or a small group of motivated individuals (private schemes). Such isolated local Community Based Health Insurance (CBHI) schemes lack the management and human resource capacities to have a significant impact on the population (2).

The survey of the three districts and the review of 182 countries regarding the UHC, both revealed that there is a high proportion of dropouts and subsidy enrollment, which is the key challenge for the sustainability of health insurance programs in Nepal. Revisiting of existing HI policy on health care packages, more choices on copayment, capacity building of enrollment assistants, and better coordination between the health insurance board and health care facilities can increase enrollment and minimize dropout (27, 30).

### Service quality and utilization

Although insured beneficiaries had better experience and perceptions of service quality and utilization, coverage was not as expected. The uninsured are perceiving and experiencing the SHIP differently than their counterparts. However, the trust did not differ between the two groups, which is very important for program scale-up. A similar finding was found in a survey carried out in Ghana, which has roughly a similar socio-economic status as ours, as both insured and uninsured were satisfied with care (13).

The idea of budget and financial adequacy, as well as the quality of service, is constant. According to research conducted by the National Planning Commission of Nepal (NPC-N), when the disability allowance of social security was granted only up to a particular quota, the service quality deteriorated, resulting in even the qualifying applicants (disabled) not being able to get it. Worse still, the issue of prejudice may imperil service consumption even more (31).

A hypothetical pre-post-quality-change study carried out in Wenzhou, China focusing on a multitiered copayment system that provisioned the increasing proportion of copayment at higher-tier hospitals than at primary health care (PHC) levels reflected that service quality is not only important overall but also more important to leverage quality services at the PHC level if we want to enhance the insurance scheme at all levels. Moreover, this hypothetical intervention would impact older adults and those with moderate health status more (32).

An insurance-related study in Kailali revealed that households without health insurance, low economic status, and heads with a low level of education were more likely to face catastrophic spending. The findings suggest a policy guideline in the ongoing national health insurance debate in Nepal. The government's health insurance program is currently at the expansion stage, thus, an increase in insurance coverage could financially help vulnerable households by reducing catastrophic health expenditures. The study concludes that households with insurance coverage, wealthier groups, headed by a male member, and heads with a higher level of education were less likely to suffer from catastrophic spending (6).

Another study digging out the renewal-related predictors showed that almost 64 % of the respondents were willing to renew their membership upon improved services. The primary determinants of annual membership renewal in HI are HH income, health care quality, and health service usage. Healthcare quality and service usage were two of the top three reasons for dropout. The study, however, did not differentiate between moral hazards or actual service utilization, thus demanding further studies on the health service utilization of the insured members (33). The GON had proposed that the procedure of treatment expenditure for chronic diseases for the ultra-poor be gradually included in health insurance under 2078 BS (16), which might increase enrollment.

### Premium, risk pooling, renewal, and benefits package

Even though the premium on perception did not differ between the two groups, the importance of annual renewal and perceived referral were found with high odds of favoring the insured beneficiaries. The benefits package was perceived as good, but some crucial health problems have not been included in the package, mainly kidney disease and other NCDs and related essential medicines. Moreover, the household heads (HHs) reluctant to enroll had lower ill susceptibility. As a result, both type-1 and type-2 moral hazards may increase. In type-1 moral hazard, the presence of insurance coverage may affect actions that affect an individual's probability of illness, for example, neglecting to prevent behaviors; and in type-2, the presence of insurance may also affect the amount and cost of care once illness has occurred, such as insured individuals demanding more medical care and possibly more expensive types of medical care. Here, the predictability among the clients of chronically ill or manifesting earlier signs and symptoms of such diseases may be differently dealt with than under an insurance policy, unless they are being insured on a lifetime basis. Having surety of risk will make higher chances of enrolling, thereby diminishing the utility of risk pooling (11). Furthermore, before including any medicine or healthcare in the benefits package, a process of the Health Technology Assessment (HTA) is needed, not only from a cost perspective but also to reach the UHC (34). Updating the benefits package has also been emphasized as an alternate strategy for UHC (30).

Some beneficiaries revealed their denial of risk pooling by not being willing to enroll as they are not sick. Having said that, Arrow hypothesizes that insurance requires the maximum possible discrimination of risks; pooling of unequal risks; i.e., those at higher disease risk should pay higher premiums (11). Moreover, a study carried out in Saudi Arabia showed that the risk perception of the general population differs, and so do preventive behaviors (35). The Saudi study reflected that people's first preference is to invest in real estate, followed by insurance, including property and health. It shows that disease-related risk awareness delivery may help to understand better and, thus, increase risk pooling. The preference of choice may also be implicated in the renewal system. A dropout analysis of SHIP carried out in Pokhara metropolitan showed that families living in rented houses have four times more odds of dropout than their counterparts. This may indicate that SHIP may be less preferred than paying for rent or other things (26). However, inversely, some HHs' willingness to pay the threshold for SHIP seemed to be as low as NPR 1,000 (1 US$ = 120.51 NPR) (24) indicating the inclusion groups to be identified. The current premium may have been perceived as higher due to the experience of catastrophic health expenditure recently or in the past. A study carried out in one of the same districts showed that nearly one-fifth of households (17.8%) suffered catastrophic health expenditure (CHE). However, there exists a vicious cycle, as the same study showed that insured beneficiaries are 57 % less likely (OR = 0.43, 95% CI, 0.26–0.70) to suffer from CHE than their counterparts (6).

The experience of expanding the benefits package was found to be significantly different between the groups. It may be further explored as food for thought that a study carried out in the Kailali district showed that a household having any member with a chronic disease has nearly two times (OR = 1.98, 95% CI, 1.67–2.34) going through catastrophic expenditure than without having such a disease (6). Thus, beneficiaries from such backgrounds (may be uninsured) may propose a similar benefits package to be expanded as some medicines for non-communicable diseases have already been covered in the package.

Risk pooling also indirectly inhibits a causal pathway in catastrophic payment, including reaching out to universal health coverage. A recent study carried out in China with longitudinal data for the last 15 years showed that even after reaching nearly universal health coverage, the richest-poorest gap has widened, the concentration index decreasing from −0.202 in 1991 to −0.613 in 2015, even though the authors suggest medical health insurance as a means of decreasing the gap (36). Another recent study carried out in China showed that inclusive insurance impacts positively income distribution and inclusive growth as well. More than this, such a type of policy strategy is more pronounced in rural and low-income households (37). Thus, health insurance and social security programs are very important tools not only to reach universal health coverage but also to reduce inequality of different types.

Alternatively, a study carried out among patients with lung cancer in Shanghai and other two cities in China showed that out-of-pocket expenditures (OOP) in two cities were less by more than one-third (36–40%) than that in Shanghai but there were more and better health services in those two cities. Inside the cities, employees had a lesser financial burden, compared to resident city dwellers (38). Another study from rural Rwanda shows that to pay bills, nearly half of the patients had to borrow money from family or friends, accruing an informal debt that they would have to repay, and 12% had to sell their belongings (39). These findings may suggest pondering fragmented health insurance policies for people from different geographic and socio-economic strata.

In the benefits package, in line with the findings in this study, a similar study showed that revisiting the healthcare packages and more choices on copayment may reduce dropout and increase enrollment (27). Another study that was carried out in the same districts showed that more than 90% of insured groups were willing to renew their membership and recommend a friend about HI. The study found that 61% had not sought any health services from health facilities outside the HI among insured groups (29).

Financial sustainability is the core of the insurance program. Gradually expanding risk pooling would improve CBHI's financial sustainability. Improving health service quality and the availability of medicines are the priorities to increase and sustain population coverage (40). Among other strategies and schemes, a multi-tiered copayment system is an alternative to a blanket policy of a health insurance scheme. A hypothetical study of changing the quality of services at the PHC level would be effective in increasing the insureds' compliance if different proportions of copayments were provided. Such proportions may be 50% and 10% at PHC levels and increase to 60 and 20 % at secondary level hospitals, and then 75 and 25% at tertiary level hospitals, respectively, for outpatient and inpatient services, as being practiced in Wenzhou (32). However, as revealed by an Australian study, physical deformity and dementia (both mainly affecting the older population) affect economic wellbeing and increase out-of-pocket expenditure, with women spending 13.1% more than men (41). Thus, the strategy for providing subsidies in premiums to older adults may increase service utilization and provide maximum benefit.

### Communication and behavioral aspects

Although there were no variations in the behavior of health care professionals between the two groups, communication appeared to be a key obstacle in SHIP, even when it came to programming satisfaction, coverage, and use. This may be obvious when we ignore the extraneous factors in communication and behavior between the beneficiaries and the HCPs. Furthermore, in the same line, a dropout analysis carried out in Pokhara metropolitan showed moderate odds (OR, 3.09; 95%CI, 1.01–9.49) of dropout due to unfriendly behavior of HCP (26). This may indicate that there is a sheer of background variables behind the poor beneficiary-provider communication, such as perceived opportunistic behavior of the insureds by providers and, thus, deviating toward the uninsured, long waiting time, poor patient management, and unmet expectations of the beneficiaries. The Ghanian survey also found that greater insured use of health-care services causes doctors' workloads to grow, influencing their behavior toward the insured. Similarly, in addition, the perceived opportunistic behavior of the insured beneficiaries by providers might have psychologically led to deviating toward the uninsured to treat them softly and behave rudely toward the insured ones. In addition, the long waiting queue of the insured beneficiaries may add to the jeopardized situation (13). However, another study in Nepal emphasized that the HCW's financial competing interests of attracting the insured patients at their private clinics would have guided them to treat them indifferently at HFs, as it was also found that most of the dropouts were relatively from well-off families, government employees, businessmen, migrant workers, and also poor class families (27).

Radio, newspapers, and TV were the most common sources of information about the SHI. Most of the participants were positive about the enrollment assistant and other services provided by the SHI scheme. Participants were more than 90% satisfied with the nature of changes in different aspects of health services after the SHI scheme. In contrast, in Pokhara, a sizable portion of households have left the SHI program. Lack of medications is the most frequently linked factor to discontinuing SHI, followed by rented housing, family members reporting good health, and unpleasant service provider conduct. Efforts to decrease SHI dropout must focus on addressing medicine supply difficulties and enhancing providers' conduct toward scheme holders. Rented households may experience fewer dropouts if insurance awareness is raised and includes provisions to change first contact points (26).

### Challenges, barriers, facilitative factors, and ways forward

Even though the factors like a challenging enrollment process, a lack of human resources, crowding, the underfunded state of health facilities, and HH visits by SHIP enrollment staff were not found to be significantly different between the two groups, we need to further discuss these as barriers since a systematic review carried out by Ranabhat et al. in the Nepalese context showed that the volunteer type of health insurance, itself, is one of the major challenges (12). Furthermore, the SHIP message has not reached the rural people, as a result, the public perception is poor. A little earlier finding from eastern Nepal-an inadequate awareness toward health-protection demands at the institutional level and family and community networks (42) may further interact with an inadequate awareness level on risk pooling as revealed in a systematic review (12), has created a greater challenge to SHIP. Our study discovered that the non-universal use of insurance cards and only allowing them to limited health facilities has confounded and barred even UHC. In addition to our findings, another study from Nepal revealed that unavailability of enough drugs, HCWs' unfriendly behaviors, and indifferent behavior toward insured patients in healthcare facilities so that they take services from their private clinics, were the main barriers to health insurance programs in Nepal (27). In addition, a large number of sanctioned positions at public health facilities remain unfilled. Performance management was found to be rarely practiced at the facility level, and there was a lack of incentives in place for personnel. A high demand for specialized doctors was found, and interviewees mentioned a need for further training of administrative, record, and financial staff. Self-reported engagement in private practice by the staff of public health facilities ranged from 4 to 90% (29). The increasing trend of unnecessary service utilization is the main barrier in health insurance programs (16). Hypothetical pre-post change in the quality of services of PHC level HF study in China showed that in post-change, home to HF distance was an additional quality indicator, to age (older) and health status (moderate, self-rated), which were significant factors in pre-change (32). This shows that increasing PHC services may also sustain health insurance and, thereby, the health system.

The major benefits of enrollment were considered to be general treatment and a reduction in financial burden. Economic status was described as the main barrier to enrolment. A vast majority of the respondents had been invited to enrolment, and 73% agreed to enrolment. The severity of health issues and perceived susceptibility to them were both associated with HI enrollment, although they were not significant predictors. However, peers' requests to register in HI, discussions with relatives, and family members' approval of enrolment were the most significant predictors of enrolment. These factors may be incorporated into future intervention plans for increasing enrolment in HI (43). Local governments and stakeholders can play an important role. In addition, the two groups had distinct experiences using IEC materials. Integration with an old-age allowance card might make things easier. The initiative might be aided by a mobile app, online registration, patient management, and increased insurance agent mobilization at the grassroots level. Despite the disparities in service quality and use, the two groups must have the same level of confidence. Regulatory initiatives like the Health Insurance Coordinating Committee Operation Model Procedure-2021 were prepared and accepted at the provincial and local levels. In addition, Health Insurance Model Local Level Declaration Procedure-2021 was also created and authorized in the same way. Provincial and local level ownership building and strict pharmacy and drug availability management and monitoring systems for health insurance are keys to the promotion and advancement of the Health Insurance Program (16).

During qualitative research among the health personnel involved in the delivery of health services, different experiences and obstacles when implementing SHIP in Nepal were explored. According to the study, consumers originally showed interest in the insurance program, but it was discontinued in later years due to a lack of medications, acceptable laboratory services, poor human resources, awareness, and interpersonal communication. They believe that the health insurance policy was put in place to reduce poverty and catastrophic medical costs and that it is crucial to make sure that the underprivileged can sign up for the SHIP without difficulty. Participants in SHIP primarily used the services for communicable diseases, but when SHIP was implemented in the district, visits for chronic conditions including diabetes and hypertension began to rise. The pattern of service use also saw certain adjustments. Service providers noted that participants with health insurance schemes visit health facilities in earlier stages of disease compared to those who do not have health insurance (16). In line with this, a willingness to pay for the program is substantially related to awareness of the scheme. It is influenced in part by social capital and awareness of the Community Based Health insurance initiative (43). Thus, it is evident that social cohesiveness and solidarity in local communities are vital to raising awareness of the benefits of the CBHI and, thus, in SHIP and other social security programs.

Among other alternative schemes, Ranabhat and his colleagues in a similar study argued that CBHI through co-operatives would be among the better models because of its cost-effectiveness and self-responsiveness. However, results from its piloting in a Chinese context showed mixed results. They have also proposed the mandatory and single-payer health insurance models as superior to others (44). Other alternative strategies proposed to protect from the catastrophic situation and pool resources include adequate human resources for health; an efficient and quality health-care delivery system; a mix of public and private funds, including government revenues; SHI; private insurance; developmental assistance for health (DAH); a strong information system; a balanced mix of services; and actions addressing social determinants of health (30, 45).

### Limitations

Despite these findings, the study has certain limitations as well. First, it was based on the information collected using records of the health facilities and the primary information obtained through interviews at a longer cross-section of time duration between the districts, i.e., 8 months. Thus, there might be some variations in results due to longer time intervals. Since the chosen facilities were implementing the programs, and beneficiaries were selected only during data collection periods of certain durations, there would be a selection bias and they may not represent the diversity of illnesses. Second, although providing an important impression, only a district from Terai and two from the hilly region probably do not represent the variation by territory or ecology to scale-up and so, results and, more specifically, the prediction of enrollment may be cautiously interpreted. Third, the quality of services and utilization needed to be checked with trade-offs of moral hazard, benefits package, premium, and other tools of health economics, which were beyond the scope of the study. Fourth, qualitative data were coded by a single person, thus, inter-coder reliability was not calculated. Finally, the study was unable to include the perspectives of the third party of the tri-polar insurance mechanism, the board members.

## Conclusion

Adherence to the SHIP trend has got momentum, weaker though, with enrollment and a bit sluggish dropout and poor renewal. Patient management, especially queue management, developing communication skills among the service providers, and timely reimbursement are recommended urgently to improve the SHIP. Community mobilization for insurance education, including benefits package with their regular updates, enrollment, and renewal processes, blended with prevalent disease threat and risk perception and the value of preventive measures to reduce moral hazard, are promoting factors to strengthen the program. Some alternate policy schemes and strategies based on three-tier governmental contexts, disease predictability, and pooling of unequal risks from different resources are warranted.

## Data availability statement

The datasets presented in this study can be found in online repositories. The names of the repository/repositories and accession number(s) can be found in the article/Supplementary material.

## Ethics statement

The studies involving human participants were reviewed and approved by Nepal Health Research Council (NHRC). The patients/participants provided their written informed consent to participate in this study.

## Author contributions

DP, CA, JB, and DN: study conceptualization and design. DP, JB, and DN: fund acquisition. DP, CA, KJ, KC, NP, NB, and AR: data collection. DP, CA, SP, and LA: analysis and interpretation of results and subsequent review and correction. DP, CA, SP, LA, and NP: draft manuscript preparation. All authors reviewed the results and discussions and approved the final version of the manuscript.
