# Supplementary material for: Adopting social health insurance in Nepal: A mixed study
Source: Front Public Health. 2022 Dec 15;10:978732. doi: 10.3389/fpubh.2022.978732 (PMC9798538; doi:10.3389/fpubh.2022.978732)
Supplement: Supplementary file 1 [file Data_Sheet_1.docx]

**Supplemental File**

S1. Table 1. Study variables

| **Dependent Variable** | Perception, Experiences, and Adherence of SHSP among consumers and service providers |
| --- | --- |
| **Independent Variables** | **Background variables**: Gender, Age, type of family, employment status, monthly income, ethnicity |
|  | **Experiences of Insurances**: past exposures to insurances, premium paid, number of years of experiences  **Current exposure to SHSP**: registration status, premium paid, enrollment health institution, types of services received, perception of the enrollment process, suitability of program, target population, benefit package, facilitators and barriers of services, service accessibility, availability, utilization, distance of health facility, cost of care among uninsured people, frequency of visits, health insurance renewal behaviors |

S1. Table 2. Sampling frame and sample selection

| **District** | **Health care Institutions implementing SHSP** | **Selected Health institutions** | **Number of SHSP enrollments** | **Study samples** | |
| --- | --- | --- | --- | --- | --- |
|  |  |  |  | **Quantitative** | **Qualitative** |
| Ilam | Ilam District Hospital | Pashupatinagar PHCC  Phikkal PHCC | 35399 | 220 | 2 FGDs  2 IDIs |
|  | Pashupatinagar PHCC |  |  |  |  |
|  | Mangalbare PHCC |  |  |  |  |
|  | Pyang PHCC |  |  |  |  |
|  | Phikkal PHCC |  |  |  |  |
| Baglung | Dhaulagari Zonal Hospital | Dhaulagari Zonal Hospital  Kushmisera PHCC | 26962 | 175 | 2 FGDs  2 IDIs |
|  | Galkot PHCC |  |  |  |  |
|  | Kushmisera PHCC |  |  |  |  |
|  | Burtibang PHCC |  |  |  |  |
| Kailali | Chaumala PHCC | Chaumala PHCC  Tikapur Hospital  Godagodi Hospital | 69434 | 427 | 3 FGDS  3 IDIs |
|  | Udasipur PHCC |  |  |  |  |
|  | Joshipur PHCC |  |  |  |  |
|  | Tikapur Hospital |  |  |  |  |
|  | Seti Zonal Hospital |  |  |  |  |
|  | Malakheti Hospital |  |  |  |  |
|  | Bhajani PHCC |  |  |  |  |
|  | Geta Eye Hospital |  |  |  |  |
|  | Ghodaghodi Hospital |  |  |  |  |
|  | Navajeevan Hospital |  |  |  |  |
|  | Lal Ratna Hospital |  |  |  |  |
|  | Bardagoriya Hospital |  |  |  |  |
| Total | 21 | 7 | 131795 | **814** | **7 FGDs,**  **7 IDIs** |

S1. Table 3. Description of Focus Group Discussions conducted in different districts

| **District** | **Date** | **Place/catchment area** | **Duration** | **Participants** | **Total FGDs** |
| --- | --- | --- | --- | --- | --- |
| Ilam | 13 March 2020 | Suryodaya Municipality, Phikkal PHCC | 30 minutes | 4 | 2 |
|  | 14 March 2020 | Suryodaya Municipality, Pashupatinagar PHCC | 35 minutes | 5 |  |
| Baglung | 23 October 2020 | Jaimini Municipality, Kushmisera PHCC | 1 hour | 8 | 2 |
|  | 28 October 2020 | Baglung Municipality, Dhaulagiri Hospital | 1 hour | 8 |  |
| Kailali | 21 October 2020 | Gauriganga Municipality, Chaumala PHCC | 40 minutes | 8 | 3 |
|  | 21 October 2020 | Ghodaghodi Municipality, Ghodaghodi Hospital | 40 minutes | 9 |  |
|  | 21 October 2020 | Tikapur Municipality, Tikapur Hospital | 40 minutes | 8 |  |

S1. Table 4.  Tools and Techniques for data collection

| **SN** | **Tools** | **Techniques** | **Application** |
| --- | --- | --- | --- |
| 1. | Client Exit Interview Guideline (Structured) | Face to face interviews with the consumers (both insured and uninsured) | To assess perception towards different attributes (premium level, unit of enrollment, management, health services benefit packages, transportation coverage, and copayment levels) and their levels of SHSP and adherencetos the program |
| 2. | Focused Group Discussion (FGD) Guideline | Interactive and probing discussion with services consumers | Different sessions for insured and uninsured consumers are organized to explore experiences and their perceptions |
| 3. | In-Depth Interview (IDI) Guideline | Face to face interviews with service providers and managers | To assess the experiences and perceptions towards the consumers and program.  To explore the moral hazards and related variables |

S1. Table 5. Travel expenditure and travel time to reach health institutions

| District | Amount paid (NRs) | Expenditure for travel to reach a health facility | | Total | Time (in minutes) | time to reach a health facility | | Total | p-value |
| --- | --- | --- | --- | --- | --- | --- | --- | --- | --- |
|  |  | Insured | Uninsured |  |  | Insured | Uninsured |  |  |
| Illam   (n=220) | ≤200 | 110(50.0) | 97(44.1) | 207(94.1) | ≤30 min | 83(37.7) | 74(33.6) | 157(71.4) | .88 |
|  | >200 | 5(2.3) | 8(3.6) | 13(5.9) | >30 min | 32(14.5) | 31(14.1) | 63(28.6) |  |
| Baglung (n=175) | ≤200 | 75(42.9) | 97(55.4) | 172(98.3) | ≤30 min | 71(40.6) | 93(53.1) | 164(93.7) | .53 |
|  | >200 | 2(1.1) | 1(0.6) | 3(1.7) | >30 min | 6(3.4) | 5(2.9) | 11(6.3) |  |
| Kailali  (n=427) | ≤200 | 212(49.6) | 214(50.1) | 426(99.8) | ≤30 min | 148(34.7) | 173(40.5) | 321(75.2) | .013 |
|  | >200 | 0(0.0) | 1(0.2) | 1(0.2) | >30 min | 64(15.0) | 42(9.8) | 106(24.8) |  |
| Total (n=822) | ≤200 | 397(48.3) | 408(49.6) | 805(97.9) | ≤30 min | 302(36.7) | 340(41.4) | 642(78.1) | .023 |
|  | >200 | 7(0.9) | 10(1.2) | 17(2.1) | >30 min | 102(12.4) | 78(9.5) | 180(21.9) |  |

S1. Table 6.  Distribution of Health service providers selected for IDIs

| **District** | **Working health facility** | **Position** | **Work experience** |
| --- | --- | --- | --- |
| Ilam | Phikkal PHC | ANM | 6 months |
|  | Pashupatinagar PHC | AHW | 2 years |
| Baglung | Kushmisera PHCC | HA/Focal Person | 2 years/ 6 yeas |
|  | Dhaulagiri Hospital | HA/ Focal Person | 5years/7years |
| Kailali | Chaumala PHCC | Focal Person/ SN | 5 years/3 years |
|  | Ghodaghodi Hospital | Focal person/HA | 4 years/4 years |
|  | Tikapur Hospital | Focal Person/Service provider-ANM | 5 years/2 years |

S1. Table 7. Qualitative findings from beneficiaries of different health facilities

| **S. N** | **Theme/pattern** | **District** | **Place** | **Responses** |
| --- | --- | --- | --- | --- |
| 1. | Knowledge about the SHSP | **Ilam** | **Phikkal PHCC** | Almost all the participants stated that they have heard about SHSP. |
|  |  |  | **Pashupatinagar PHCC** | The majority of the participants knew about the SHSP program. And the uninsured participants had also heard about SHSP. |
|  |  | **Baglung** | **Kushmisera PHCC** | Cent percent of the participants were known about SHSP. |
|  |  |  | **Dhaulagiri Hospital** |  |
|  |  | **Kailali** | **Chaumala PHCC** | The majority of the participants had heard about the SHSP while some of them were in confusion between private insurance and SHSP. One of the participants stated “I have heard about SHSP but I don’t know much about it. Similarly, another participant opined “if we can have insurance card with us, we can get services from every government health institution up to 50,000.” In the meathe ntime, majority of uninsured participants had no or minimal knowledge about SHSP. |
|  |  |  | **Ghodaghodi Hospital** | The majority of uninsured participants didn’t know about SHSP and some of the participants despite being insured also did not know SHSP. |
|  |  |  | **Tikapur Hospital** | Almost half of the participants were aware of SHSP whereas half of them were unaware of SHSP. Some of them stated “SHSP saves health care costs and it is extremely useful when someone finhas ancial hardships. “If health insurance is done, treatment can be done ata minimal cost. |
| 2. | Utilization of SHSP | **Ilam** | **Phikkal PHCC** | Majority of the participants enrolled in SHSP from the initial years of SHSP. One of the participants stated “I got enrolled in the SHSP since its inception in the year 2072BS.” Similarly, another participant said that he had received the services under SHSP schemes when he had motorbike accident. Almost all the participants accepted that some of the SHSP insured discontinued because they the services were not efficiently delivered. |
|  |  |  | **Pashupatinagar PHCC** | The participants unitedly reported that “people have been enrolling in the SHSP in different years since the start of program and their enrollment has been increasing; however, discontinuity rate is also high among insured ones.” One of the insured participants who had insurance under SHSP reported that he had undergone for surgery for gall stone. He added that he was satisfied with services and the referral system. On the other hand, one of the insured participants added “I am quite unsatisfied by the services as the service provider’s way of dealing with the public was not appropriate.  Therefore, I had discontinued the SHSP program.” |
|  |  | **Baglung** | **Kushmisera PHCC** | About half (50%) of the participants enrolled in SHSP before 2 years ago and more than 80 percent who were enrolled in SHSP are satisfied with services  Some uninsured participants quoted “we did not enroll in SHSP due to lack of money” and some added “We are not sick, so why do we need SHSP” |
|  |  |  | **Dhaulagiri Hospital** | About 50 percent population were enrolled in SHSP before 3 years ago and the enrollment practices have been improving in recent years.  Enrolled people have received services provided under SHSP. In the meantime, more than 60percent insured population were not satisfied with services management SHSP at Dhaulagiri Hospital. Some participants added “some people received service for the first time and thereafter they seldom received services due to due to perceived poor quality of service and its management.” |
|  |  | **Kailali** | **Chaumala PHCC** | Participants reported that many people enrolled in the SHSP starts to decline in the subsequent years because of the low satisfaction. Among the insurers, they have received services for different ailments such as headache, fever, common cold, backache through the SHSP schemes. In addition, they were privileged with eye checkup and speculation, treatment of gall bladder stone, joint problem, X-ray, Video X-ray, gastritis etc. through the SHSP schemes. |
|  |  |  | **Ghodaghodi Hospital** | Participants stated that majority of the consumers were enrolled in the SHSP from the inception of SHSP and they are continually enrolling into SHSP. Those who did not enrolled in SHSP, they were due to Poor financial status, no knowledge about SHSP and even they did not have renewed insurances. |
|  |  |  | **Tikapur Hospital** | Some of the participants were recently enrolled whereas some of them were enrolled 3 to 4 years back. Majority received common services like X-rays, blood tests, laboratory tests, treatment of common illnesses like fever, headache, common cold from SHSP implemented health care institutions. One of the participants said “My mother is suffering from a kidney-related problem but the treatment is not available through SHSP.” |
| 3. | Perception of SHSP | **Ilam** | **Phikkal PHCC** | Some opined that the premium charged is very reasonable but some stated that it is expensive.  In the meantime, one of the participants stated. “It is the total waste of money. My family doesn’t get chances to utilize that amount.”  Almost all participants opined that the household/family size as the unit beneficiary for SHSP. More than half of the participants noted “SHSP is a good approach where the health of every family member is secured.” Furthermore, it is perceived as very supportive in health care; nevertheless, it is sometimes a problem for a joint family with members more than 10.  One of the participants opined “The concept of family as a unit is good. Rather than focusing on only one member, taking family as a is comparatively good.”  In regards to the management of services, one of the participants reported that he visited health institution three times for the treatment of a single disease but couldn’t. At the different times, shortage of equipment and health personnel were reported that decreased the quality of services. Therefore, he perceived that the management of services was poor.  On the other hand, some participants opined that almost 70-80 percent of health care costs are covered by SHSP.  One of the participants expressed that the coverage of care under SHSP has been increasing since its inception. He quoted “Once a villager whose treatment cost for a disease was about Rs 50,000, about Rs 40,000 cost was borne by the insurance. This incident increased the enrollment and attracted people towards SHSP.” |
|  |  |  | **Pashupatinagar PHCC** | Majority of the insured and uninsured participants opined that the premium charge is fair and affordable. They further added, it would be better if the premium cost would be borne by the governmental bodies in the case of marginalized people or those who face financial hardship.  Some others stated that “the premium is little expensive and it would be more appropriate if the amount is around Rs 1000 instead of paying Rs 3500 annually.”  All agreed that family as a unit of service for SHSP is quite good concept. Some of them argued that if the family members up to 6 are considered as unit, that constitutes very sound as it can have their parental inclusion in SHSP.  The major complaints put forward by the people regarding the management of SHSP at the health facility were the unavailability of the health equipment, no fluent delivery of services, waiting in long-long queues, etc.  In regards to the management of services, participants reported that “We can’t get any services that we are seeking for. Very few services are included in SHSP.”  So far as concerned with the coverage, almost 80 percent of the population have access to SHSP services and door to door visits made by insurance agents made possible to improve enrollments in SHSP. In the meantime, uninsured participants said that the coverage of the SHSP is low and services are not satisfactory. One of the uninsured participants quoted “It has neither covered a large population nor has the awareness regarding SHSP reached the rural parts of Nepal.” |
|  |  | **Baglung** | **Kushmisera PHCC** | Majority of the participants stated that “If we fall sick, then there is increase in expenditure for treatment, so SHSP is good while in case of no sickness, it is waste of money.” All participants agreed that the family as unit of service good as it covers the health care for all family members.  Management of services has been improved however, it is necessary to expand the number of services like ultrasound, laboratory and specialization services at the PHCC level. Due to limitations of service packages availability, people opt higher health care institutions for the care.  One of the participants stressed “All medicines are not available; all diseases are not treated and there is no coverage for expensive medicine.” |
|  |  |  | **Dhaulagiri Hospital** | Participants stated that the premium charged for SHSP is reasonable; however, reduction or waiver of this for poor people could enhance the large population coverage.  They further added that the family as unit of service is good concept.  Some of the participants stressed that the management of SHSP of the health facility is not satisfactory. “When we go to hospital, they ignore the patients and say there is no medicine.  Some of the participants stressed that “nature of diseases and health problems are not known in advance. So, the type of services covered under the SHSP does not meet the needs of health care and they have to seek care from other health care institutions.” One of the participants expressed that “it is good in paying NRs per family pertains coverage of 1 Lakh. Meanwhile, some participants opined that those who were not insured, it was because ignorance or perceived lack of services of patients at the SHSP implemented health care institutions. |
|  |  | **Kailali** | **Chaumala PHCC** | Most of the participants stated that premium amount charged for SHSP is very affordable for those who frequently fall sick. One of them stated “if somebody does not have disease, it is the waste of money.”  All the participants reported that family as the unit of service or the individuals as unit also constitutes good. Long waiting time, limited coverage and availability of services are management issues identified in SHSP; nonetheless, majority of the population are satisfied with SHSP services. Benefit package under the SHSP is limited and need to expand the services. Sometimes, the insured individuals have to purchase medicines from private health institutions because it is not covered in this scheme. All unitedly expressed that there was high coverage and high dropout rate. |
|  |  |  | **Ghodaghodi Hospital** | Participants stated that the premium amount is appropriate and affordable for those who avail treatment; however, it is waste of money if we don’t fall sick. Similarly, family as the unit of service is a good concept.  Insured people stated “Service providers pay less attention to the insured people than the uninsured people and Services are not provided in time to the insured ones.”  Therefore, despite being enrolled in SHSP, people preferred services from private health institutions. |
|  |  |  | **Tikapur Hospital** | Majority of the participants reported that premium amount charged for SHSP was reasonable for family and it would be useful if the it would be covered by the government in case of disadvantaged population.  Meanwhile, some of the participants stressed that premium amount charged for SHSP was expensive along with the renewal system was not user friendly. Many participants stated that “It is not a good concept to charge the same premium for a family with few or more (up to 5 members) number of members. There should be different provisions for individuals or multiple family members. Majority of the participants stated that the benefit package under SHSP demotivate. One of the participants quoted “The package is demotivated. I once extracted a tooth but there is no provision for replacing that tooth with new one.”   Majority of the participants reported that the coverage of SHSP in the catchments area of Tikapur Hospital, Tikapur Municipality was almost 70 percent while few of them perceived the low coverage of SHSP. |
| 4. | Private sector involvement | **Ilam** | **Phikkal PHCC** | All the participants reported that there were no involvements of private institutions observed in SHSP delivery in Suryodaya municipality, Ilam. |
|  |  |  | **Pashupatinagar PHCC** | There was no involvement of private sectors in SHSP delivery.  However, some of the private health institutions providing certain information and referring centers. |
|  |  | **Baglung** | **Kushmisera PHCC** | There were no involvements of private sectors in SHSP service delivery. |
|  |  |  | **Dhaulagiri Hospital** | There were no involvements of private sectors in SHSP service delivery. |
|  |  | **Kailali** | **Chaumala PHCC** | We haven’t noticed involvement of private sectors in SHSP. |
|  |  |  | **Ghodaghodi Hospital** | People are not aware of the fact that SHSP bears the cost of various medicines and it is also delivered from some of the private health institutions. Despite being enrolled in SHSP, people prefer services from private clinics. |
|  |  |  | **Tikapur Hospital** | The majority of the participants had no idea of involvement of private sectors in SHSP delivery. Nevertheless, few of them told that there was No involvement of private sectors in SHSP. |
| 5. | Perceived promoting factors | **Ilam** | **Phikkal PHCC** | Participants opined that community-level awareness and motivated to peripheral level can promote the utilization of services. One of the participants quoted “Being a teacher, I have told every student about the SHSP program of Nepal government.” Similarly, “I being the first person to be enrolled in SHSP, I have made my community aware about it and made them enroll in the program.” |
|  |  |  | **Pashupatinagar PHCC** | Placement of citizen charter regarding SHSP, community and group awareness program could promote utilization of SHSP.one of the participants stressed “Change in public perception is mostly necessary.”  Similarly, two participants added “Government should give all the information about this program to all sections of populations including marginalized people.” |
|  |  | **Baglung** | **Kushmisera PHCC** | Public awareness, good services from government, public friendly environment, availability of services are the factors promoting utilization of SHSP services. |
|  |  |  | **Dhaulagiri Hospital** | Public awareness and availability of all services in hospital could promote the SHSP. |
|  |  | **Kailali** | **Chaumala PHCC** | Encouraging the uninsured people to have insurance, timely follow up for renewal, timely delivery of services and public awareness could promote the SHSP. One of the participants stressed that “Stakeholders must encourage uninsured to get enrolled in SHSP.” |
|  |  |  | **Ghodaghodi Hospital** | Increase in the service package such as inclusion of more services items and medicines under SHSP, orienting people and engaging local authorities could be useful to promote SHSP. |
|  |  |  | **Tikapur Hospital** | Addition of service package, promoting awareness among populations, orienting the benefits of service package under SHSP, mobilizing FCHV’s to inform people about SHSP could be effective strategies to promote SHSP. One of the participants reported that “People are mostly unaware about the insurance and those are insured, they have no proper idea in the process of getting enrollments and service use under SHSP.” |
| 6. | Suggestions for improvement | **Ilam** | **Phikkal PHCC** | Insurance agent’s role is crucial to improve the coverage. Extending the services to private institutions  Routine monitoring and tracking They further added that “The services should not be limited to minor diseases only. They should be more focused at treating major NCDs too.” In the meantime, one of them shared the experience “The people who are currently living in Kathmandu but enrolled from Phikkal PHCC cannot avail service under SHSP and they cannot come to Ilam for renewal or referral cards. Due to such conditions dropout rate is also increased and motivations for SHSP is decreased” They also opined that developing user-friendly referral mechanism and availing the services from all health intuitions promote the SHSP.” |
|  |  |  | **Pashupatinagar PHCC** | Advertisement of SHSP program in wider dimension, paying equal attention to both the insured and uninsured people, training to the health workers, social leaders and orienting the local people could promote the SHSP. |
|  |  | **Baglung** | **Kushmisera PHCC** | Improving the service delivery in terms of timely provision of care, increasing more hospitals/health institutions, expanding services and availing more types of services from PHCCs could be useful in expansion of SHSP. |
|  |  |  | **Dhaulagiri Hospital** | Publishing all services in citizen charters and developing the system for timely delivery of services could enhance the SHSP. |
|  |  | **Kailali** | **Chaumala PHCC** | Providing mass awareness on periodic basic regarding SHSP, addressing the genuine feedbacks of consumers, conducting orientation programs, minimizing carelessness during service provisions, upgrading service packages and provision of insurance free of cost for underprivileged could promote the SHSP. |
|  |  |  | **Ghodaghodi Hospital** | Increasing awareness and improving the management of health institutions for effective delivery of SHSP services could be useful strategies for promotion of SHSP. |
|  |  |  | **Tikapur Hospital** | Improving the care provider’s response towards the consumers’ needs could be useful because the arrogant behavior of stakeholder’s providers motivated people to enroll in SHSP.” |

S1. Table 8. Qualitative findings from program managers and service providers

| **S. N** | **Theme/pattern** | **District** | **Place** | **Responses** |
| --- | --- | --- | --- | --- |
| 1. | Coverage of SHSP | **Ilam** | **Phikkal PHCC** | Almost 60-70 percent population were insured under SHSP scheme.  Insurance agents of the respective wards facilitate the consumers. These agents visit rarely in the community and their lack of motivations in the program limited the further expansions of SHSP and its acceptance. |
|  |  |  | **Pashupatinagar PHCC** | Approximately 80 percent people are insured and higher enrollments were observed from ward number 2 and 3 of Suryodaya Municipality were mainly and Janajati were mainly insured. |
|  |  | **Baglung** | **Kushmisera PHCC** | More than 70 percent population attending PHCC were found to be insured under SHSP. Insurance agents of the respective wards facilitate the consumers; however, their frequency of visit and follow up was low. |
|  |  |  | **Dhaulagiri Hospital** | About 50-90 percent population who attended PHCC for services were insured under SHSP and most of the population of wards 1 and 5, Jaimini Municipality mainly utilized (>90%) the SHSP services. Furthermore, almost all the people with chronic diseases have had made health insurances. |
|  |  | **Kailali** | **Chaumala PHCC** | There is increased coverage of SHSP in recent years and almost 50-70 percent population have been insured. |
|  |  |  | **Ghodaghodi Hospital** | Patients from Bajani, Malakheti, Joshipur have utilized the services at highest possible. Overall, the coverage of SHSP is almost around 70 percent. |
|  |  |  | **Tikapur Hospital** | "Initially it was very good but, in the middle, there was the scarcity of medicines which resulted in decrease in the number of insured people. However, the coverage is increasing now." |
| 2. | Perceived evaluation of SHSP | **Ilam** | **Phikkal PHCC** | Existing premium rate with NRs3500/per family with 5 people and additional 700 for adding family members, services up to 1 lakh was perceived suitable among the consumers as they affirmatively report it. However, availability of limited services sometimes makes questionable about the premium. The SHSP services were perceived to be very cheap when it is compared with private sectors services and the services were mostly used by insured people as compared to uninsured ones. Insured people mainly with NCDs (mainly hypertension) utilized services of SHSP.  Community-acceptance was improved with 80-90 percent people are satisfied with services available at health institutions. Since its inception, there has been limited availability of budget and there was difficulty in accessing medicines and purchasing them. The referral center was limited to few hospitals only. |
|  |  |  | **Pashupatinagar PHCC** | Coverage of the service has been improving; however, there was limited availability of services under SHSP. Service utilization among insured population was higher than those uninsured population and insured people who had hypertension had mostly utilized services of SHSP.  Except exceptional cases, in most of the states the community acceptance of SHSP was good. There were problems in accessing and purchasing the equipment and medicines |
|  |  | **Baglung** | **Kushmisera PHCC** | The existing premium is appropriate and affordable and there were no disappointments observed among population in regards to the premium amount and it was considered the cost-effective mechanism of service coverage for all population.  Community acceptance was excellent as there was almost 80-90 percent population are observed to have satisfaction with SHSP. Nevertheless, referral system was limited to few hospitals which restricted the access to services. It was perceived to be cost effective, economic and user friendly and it was far economic than all private health care institutions. Referral system is still limited to some hospitals only due to which it limited the public access to care. Patients referred to higher centers have to be in queue for the care in higher health care institutions because of this, some insured people also discontinued. |
|  |  |  | **Dhaulagiri Hospital** | Limited services were availed under SHSP in relation to the public expectations. Nevertheless, utilization of services among insured was higher than that of uninsured patients and the patient of hypertension and other noncommunicable diseases higher tendency to utilize SHSP services. Community acceptance of the SHSP was increased; and both the enrollment and renewal were also improved.  The program was implemented with the availability of limited resources since its inception. Premium charged for the SHSP was publicly perceived to be appropriate and affordable and most of the consumers were satisfied with SHSP services.  Almost 80-90 percent population attending hospital for care were satisfied with services. Referral system was limited to selected hospital only. Therefore, it could not be user friendly. |
|  |  | **Kailali** | **Chaumala PHCC** | All the available services under SHSP were provided to the consumers as per the system and insured people observed to fully utilize the services. He further stated that "Many people still do not have awareness about the SHSP and some old age people expect the SHSP services with old age allowance card." Community acceptance has also been improving and some people exemplify "if a person simply falls sick his health care cost around in a year is likely to be 2000/3000 for common illnesses and likewise if this cost is used in enrolling in SHSP, we can get services up to 1 lakh in a year then, why not to get enrolled in SHSP." |
|  |  |  | **Ghodaghodi Hospital** | A focal person of SHSP reported that the emergency and referral services are provided from this hospital and mostly insured patients attend to get referral for higher center.  Community acceptance of the program was low because of lower level of awareness among population. |
|  |  |  | **Tikapur Hospital** | Service providers of the Tikapur Hospital experienced that the accessibility and availability of all kinds of services are limited. therefore, some patients might have suffered. Annual renewal system of SHSP was not perceived to be user friendly due to which there was poor adherence and high proportion of drop out. |
| 3. | Providers’ perception toward consumer behaviors | **Ilam** | **Phikkal PHCC** | Uninsured people stated that limited services are available and if there are no illnesses through the year, it is waste of resources. On the other hand, insured people are satisfied with SHSP services and sometimes it is difficult to avail services in time and referral system is also felt hectic. |
|  |  |  | **Pashupatinagar PHCC** | Insured people sometimes feel irritated to wait in queue to receive services. Both insured and uninsured face some problems during high patient flow. |
|  |  | **Baglung** | **Kushmisera PHCC** | Insured people perceived that it has supported very well to avail health care. Some perceived that if the insurance schemes were not launched, we might not be service because we could not be able to spend for chronic disease care and continuous medication. |
|  |  |  | **Dhaulagiri Hospital** | In majority, the community people's attitude towards SHSP was positive. Some people perceived that they become disappointed themselves when uninsured got limited services than those insured consumers. |
|  |  | **Kailali** | **Chaumala PHCC** | Insured people have good availability and coverage of care. |
|  |  |  | **Ghodaghodi Hospital** | It was positively perceived among insured and uninsured population. |
|  |  |  | **Tikapur Hospital** | Some insured people claim that "We have done insurance; we must be treated first and referral must be done based on our preference. They further stressed that the services must be made available even from private institutions. |
| 4. | Problems faced by the insured and uninsured consumers | **Ilam** | **Phikkal PHCC** | It was perceived to be high economical loss having no SHSP enrollments. Insured consumers have perception of difficult in dealing with hectic referral system. |
|  |  |  | **Pashupatinagar PHCC** | Unavailability of some medicines under SHSP takes long time to bring and make medicines available. |
|  |  | **Baglung** | **Kushmisera PHCC** | It was perceived to be high economical loss having no SHSP enrollments. Insured consumers have perception of difficult in dealing with hectic referral system and long waiting time. |
|  |  |  | **Dhaulagiri Hospital** | Unavailability of some medicines under SHSP takes long time to bring and make medicines available. Furthermore, uninsured people perceived that there was expensive treatment without SHSP. Insured consumers have perception of difficult in dealing with hectic referral system. |
|  |  | **Kailali** | **Chaumala PHCC** | Frequent complaints of the consumers were the limited availability of services from SHSP implemented health institutions. |
|  |  |  | **Ghodaghodi Hospital** | Service provider opined that "It is only their feeling but there are no any problems they have to face. He further added "Both the insured and uninsured have to wait for their checkup time to come in queue if the patient flow is high." |
|  |  |  | **Tikapur Hospital** | Consumers attending hospital have not complained so far.  He quoted, "I have not observed any problems faced in receiving services as it is very systematic. There were no verbal complaints as well as written feedbacks from public and consumers in regards to the problems faced" |
| 5. | Providers’ perception toward underprivileged groups in SHSP | **Ilam** | **Phikkal PHCC** | There should be effective delivery of services at any time with increased concentration on underprivileged people. Underprivileged people will be ensured if the government will manage their premiums and making it compulsory. |
|  |  |  | **Pashupatinagar PHCC** | Frequent visits by insurance agents to every HHs of low socioeconomic status (SES) and making this compulsory for all. |
|  |  | **Baglung** | **Kushmisera PHCC** | Making compulsory provisions of SHSP and waiving their premium and renewal fees could promote the enrollments of underprivileged class population. Although, there was provisions to encourage the people for enrolments, many disadvantaged class populations did not insure because of poor knowledge of SHSP. Therefore, supportive mechanisms for service delivery and local level awareness campaign should be done for greater engagements. |
|  |  |  | **Dhaulagiri Hospital** | The SHSP schemes are free for special groups however, their enrollment is still low. All kinds of services should be free of cost for them because they seldom utilize service with the fear of additional expenditures. |
|  |  | **Kailali** | **Chaumala PHCC** | Insurance free of cost for underprivileged could promote SHSP among these populations. . |
|  |  |  | **Ghodaghodi Hospital** | "Respective municipality must enroll underprivileged groups into SHSP rather than providing them with relief fund." |
|  |  |  | **Tikapur Hospital** | Frequent visits by insurance agents at the household level, discount on premium or free of cost and spreading awareness to the people about the services and benefits that are available through SHSP effectively lead to the success of SHSP among low socioeconomic class populations. |
| 6. | Problems faced at institutional level | **Ilam** | **Phikkal PHCC** | There were limited human resources, service packages and financial resources in relations to high patient flow. Sometimes, when the patient's expectations of the specialized services are not met, they make verbal abuses/irrelevant quires and develop the negative attitudes towards SHSP. |
|  |  |  | **Pashupatinagar PHCC** | Limitations in human and financial resources, limited availability of services and medicines and nagging from public at times of slow supply of services were the frequent problems encountered at the time-of-service delivery. |
|  |  | **Baglung** | **Kushmisera PHCC** | Inadequate internal resources and limited human resources in relations to the high patient flow at the PHCC constraints the service delivery under SHSP. |
|  |  |  | **Dhaulagiri Hospital** | Low availability of human resources, limited availability of medicines and supplies at the hospital led the poor management of services. There were increased patient flow under SHSP scheme but due to limitations in service providers make them to wait for long period of time. This resulted in poor acceptance or high discontinuity rate among insured people. |
|  |  | **Kailali** | **Chaumala PHCC** | There were no major problems encountered at the institutional level in delivery of SHSP; however, the insured people frequently report their needs remain unmet as the services are limited under SHSP. |
|  |  |  | **Ghodaghodi Hospital** | Delayed reimbursement of expenditures for the long period of time resulted into poor performance in service delivery and face financial crises periodically. |
|  |  |  | **Tikapur Hospital** | Dealing with the rude behaviors of patients in case of differences in their expectations and existing state of service delivery make the unusual and unpleasant environment. He further added " many patients do not have idea of SHSP and they attend in hospital with expectations all free services. If their services are out of insurance coverage, they become annoyed and badly scold the service providers" |
| 7. | Occupational risks experienced during service delivery | **Ilam** | **Phikkal PHCC** | Provided the services to infectious patients without personal protective equipment (PPE) such as gloves and masks. |
|  |  |  | **Pashupatinagar PHCC** | Encountered the problems while at the time of injecting canula due to unavailability of equipped materials. |
|  |  | **Baglung** | **Kushmisera PHCC** | Expectations of patients is sometimes not met and they become uncooperative during service provisions. In such cases, provision of care without PPE threatens for occupations hazards. |
|  |  |  | **Dhaulagiri Hospital** | Encounter some hazards due to unavailability of equipped materials. |
|  |  | **Kailali** | **Chaumala PHCC** | Encounter some hazards due to unavailability of equipped materials. |
|  |  |  | **Ghodaghodi Hospital** | Risk of transmission of infectious diseases, communicable diseases like HIV, hepatitis were experienced due to lack of PPEs |
|  |  |  | **Tikapur Hospital** | No occupational risk experienced. |
| 8. | Facilitators of program management | **Ilam** | **Phikkal PHCC** | Regular monitoring of program, regular supply of services and medicines, good inventory management, monthly progress reporting and review meeting, distribution of responsibilities among the staff had facilitated the management of SHSP services at PHCC. |
|  |  |  | **Pashupatinagar PHCC** | Good store management practices and appropriate handling of health care waste supported to promoted SHSP management. |
|  |  | **Baglung** | **Kushmisera PHCC** | Regular monitoring of program, regular supply of services and medicines, good inventory management, monthly progress reporting and review meeting, distribution of responsibilities among the staff and contingency meetings for the immediate issues facilitated the management of SHSP. |
|  |  |  | **Dhaulagiri Hospital** |  |
|  |  | **Kailali** | **Chaumala PHCC** | Regular involvement of the staff in the delivery of services to insured and uninsured people in usual fashion gradually established and managed the program. |
|  |  |  | **Ghodaghodi Hospital** | Program was run as usual with other services. |
|  |  |  | **Tikapur Hospital** | Timely engagement of the staff and their shared responsibilities for the delivery of services were key factors in the management of services. |
| 9. | Measures to improve coverage, accessibility and implementation modalities of SHSP | **Ilam** | **Phikkal PHCC** | Mobilization of insurance agents to encourage the people at local level for enrollment and utilization of services under SHSP could be useful to promote the different dimensions of SHSP. |
|  |  |  | **Pashupatinagar PHCC** | Making wider publicity through reports, media, meetings, pamphlets and interaction sessions including experience sharing could be useful to promote SHSP. |
|  |  | **Baglung** | **Kushmisera PHCC** | Mobilization of insurance agents to encourage the people at local level for enrollment and utilization of services under SHSP could be useful to promote the different dimensions of SHSP. In addition, expansion of services to all the public and private health institutions could gradually improve the coverage, accessibility and utilization of services. |
|  |  |  | **Dhaulagiri Hospital** |  |
|  |  | **Kailali** | **Chaumala PHCC** | Providing the information to the public at the time of their visits at health institutions and mobilization of insurance agents at all the grass root level ultimately improve the coverage of SHSP. |
|  |  |  | **Ghodaghodi Hospital** | Spreading awareness through FCHV's and working in coordination with local bodies could improve the coverage; and the health institutions could discharge their responsibilities at par their job descriptions. |
|  |  |  | **Tikapur Hospital** | Promoting public awareness, developing effective referral mechanism, encouraging individuals/households/villagers/tole residents or specific groups could improve the coverage of SHSP. |
| 10. | Ways forward | **Ilam** | **Phikkal PHCC** | Increasing the human resources, training of them and their effective mobilization could overcome the barriers of SHSP. |
|  |  |  | **Pashupatinagar PHCC** | Mobilization of trained and experienced staff and their retention could enhance the SHSP. |
|  |  | **Baglung** | **Kushmisera PHCC** | Increase in human resources, training of service providers development of online management system, use of Mobile App, improving the public awareness to reduce the rumors, effective mobilization of insurance agents at grass root level, orienting people, interacting with local stakeholders and linking the service providers, social leaders and school teachers for SHSP promotion will overcome the barriers of SHSP. |
|  |  |  | **Dhaulagiri Hospital** | Placement of trained staff, their retention, use of technology for information management, increased in number of service providers and service sites gradually promote the SHSP. |
|  |  | **Kailali** | **Chaumala PHCC** | Establishing the public trust through effective and efficient mechanism ultimately reduces the rumors and barriers. Prompting the public relations through provider- community interactions could sustain the SHSP. |
|  |  |  | **Ghodaghodi Hospital** | "If there is timely reimbursement then, satisfaction level among consumers will be high and as a result coverage will also increase." |
|  |  |  | **Tikapur Hospital** | Development and implementation of mechanism for timely reimbursement of expenditure claims could enhance the SHSP services. |
